# Downregulation of GeBP-like α factor by *MiR827* suggests their involvement in senescence and phosphate homeostasis

**DOI:** 10.1186/s12915-021-01015-2

**Published:** 2021-05-03

**Authors:** Chao Ma, Qiuju Chen, Shiping Wang, Amnon Lers

**Affiliations:** 1 Department of Plant Science, School of Agriculture and Biology, Shanghai Jiao Tong University, Shanghai, 200240 China; 2 Department of Postharvest Science, Agricultural Research Organization, Volcani Center, HaMaccabim Road 68, 7505101 Rishon LeZion, Israel

**Keywords:** Senescence, *Arabidopsis thaliana*, miRNA, *miR827*, *GPL*, Phosphate homeostasis

## Abstract

**Background:**

Leaf senescence is a genetically controlled degenerative process intimately linked to phosphate homeostasis during plant development and responses to environmental conditions. Senescence is accelerated by phosphate deficiency, with recycling and mobilization of phosphate from senescing leaves serving as a major phosphate source for sink tissues. Previously, *miR827* was shown to play a significant role in regulating phosphate homeostasis, and induction of its expression was also observed during Arabidopsis leaf senescence. However, whether shared mechanisms underlie potentially common regulatory roles of *miR827* in both processes is not understood. Here, we dissect the regulatory machinery downstream of *miR827*.

**Results:**

Overexpression or inhibited expression of *miR827* led to an acceleration or delay in the progress of senescence, respectively. The transcriptional regulator GLABRA1 enhancer-binding protein (GeBP)-like (*GPLα*) gene was identified as a possible target of *miR827*. *GPLα* expression was elevated in *miR827*-suppressed lines and reduced in *miR827*-overexpressing lines. Furthermore, heterologous co-expression of pre-*miR827* in tobacco leaves reduced *GPLα* transcript levels, but this effect was eliminated when pre-miR827 recognition sites in *GPLα* were mutated. *GPLα* expression is induced during senescence and its inhibition or overexpression resulted in senescence acceleration and inhibition, accordingly. Furthermore, *GPLα* expression was induced by phosphate deficiency, and overexpression of *GPLα* led to reduced expression of *phosphate transporter 1* genes, lower leaf phosphate content, and related root morphology. The encoded GPLα protein was localized to the nucleus.

**Conclusions:**

We suggest that *MiR827* and the transcription factor GPLα may be functionally involved in senescence and phosphate homeostasis, revealing a potential new role for *miR827* and the function of the previously unstudied GPLα. The close interactions between senescence and phosphate homeostasis are further emphasized by the functional involvement of the two regulatory components, *miR827* and GPLα, in both processes and the interactions between them.

## Background

Leaf senescence is a genetically controlled degenerative process that leads to cell death [1–3]. During senescence, the cellular structure, metabolic activities, and physiological role of the leaf are greatly altered. Chloroplasts degenerate and the photosynthetic apparatus disassembles [4, 5]. Biochemical processes that occur at the onset of senescence include altered gene expression, macromolecule degradation, membrane destabilization, and fluctuations in hormone levels [3, 6]. It is suggested that during the evolution of senescence, different biochemical, cellular, integrative, and adaptive systems were progressively added to the ancient primary core process as the evolving plant encountered new environmental and developmental contexts [7]. Senescence occurs as part of normal development; however, it can be induced prematurely by stressful environmental stimuli [8, 9].

To cope with nutrient-deprived environments, plants must sense changes in external and internal mineral nutrient concentrations and adjust their nutrient metabolisms to meet the demands of plant growth [10]. In annual plants, including *Arabidopsis*, leaf senescence is highly responsive to nutrient stress. A limited supply of micro- or macronutrients can induce senescence in leaves in order to mobilize resources, such as inorganic phosphate (Pi) and nitrate, to the younger sink organs, thereby ensuring reproduction and contributing to nutrient-use efficiency [11–16]. The leaf is a sink for nitrogen (N) and mineral nutrients during the early stages of development and becomes a nutrient source once it begins to senesce. Senescence involves complex metabolic changes to enable nutrient recycling and allocation [17–19]. Nutrient salvage from older leaves has the adaptive value of recycling nutrients that may be limiting in the environment or that are energetically costly to acquire [14, 17, 20].

Senescence is characterized by significant changes in gene expression patterns [21, 22], including repression of genes, such as those associated with photosynthesis, and induction of others, including senescence-associated genes (SAGs) that participate in different metabolic and regulatory aspects of senescence [22, 23]. The available knowledge about senescence regulation clearly suggests that it is composed of a complex of pathways forming a network responsible for activation of the different SAGs [1, 24, 25]. At least some of the SAGs’ regulation occurs at the transcriptional level [26–28]. The complexity of senescence regulation is also demonstrated by the involvement of a vast number of different transcription factors from different families [24, 29]. The involvement of microRNAs (miRNAs) in senescence regulation has been recently reviewed [30, 31]. In *Arabidopsis*, *miR164* was identified as a key player in senescence through control of its target, the senescence transcriptional activator gene *ORE1/AtNAC092* [32]. *MiR319* was also shown in *Arabidopsis* to positively regulate leaf senescence by regulating jasmonic acid biosynthesis [33]. Additional studies suggest the involvement of other miRNAs in senescence [34–36]. We previously conducted large-scale analyses to identify senescence-inducible miRNAs [37]. Many of the identified miRNAs were reported to be involved in nutrient responsiveness, which is consistent with the nutrient-remobilization process taking place during senescence. Among these, *miR827* was found to be significantly induced during leaf senescence, its expression peaking in late senescence [37]. *MiR827* was shown to play significant roles in regulating Pi homeostasis in plants in a nitrate-dependent fashion [38–41]. Pi deprivation results in induction of *miR827* expression, which post-transcriptionally represses transcript accumulation of its target gene nitrogen limitation adaptation (NLA) [39, 42]. NLA can mediate the ubiquitination and degradation of the plasma-membrane-localized phosphate transporter 1 (PHT1) family of Pi transporters, thereby affecting Pi homeostasis [39].

Maintenance of Pi homeostasis is crucial for crop production [43, 44]. Low levels of available Pi in the soil limit biomass and yield potential, and therefore, remobilization of Pi within the plant is important [44, 45]. Pi remobilization from senescing leaves is a major source of Pi for sink tissues. While it is clear that plants are efficient at recycling Pi from senescing leaves, our knowledge of the molecular components involved in the process is limited compared to studies of N remobilization [46, 47]. Plants can remobilize over 50% of the Pi from senescing leaves [48]. Thus, translocation of Pi from older to developing leaves is quantitatively important. Pi remobilization and transportation are effected by the action of hormones, transcription factors, and Pi-scavenging enzymes [47]. Pi transporters are critical for Pi allocation and remobilization within plants [49–51]. The *Arabidopsis* Pi transporter PHT1;5 mobilizes Pi between source and sink organs and influences the interaction between Pi homeostasis and ethylene signaling [50]. PHT1;5 overexpression resulted in altered Pi remobilization and premature senescence. PHT4;6 was also found to be involved in senescence-associated processes in *Arabidopsis* [52].

In this report, the close interactions between senescence and Pi homeostasis are further emphasized by the functional involvement of two regulatory components: *miR827* and the GLABRA1 enhancer-binding protein (*GeBP*)-like regulatory factor *GPLα*, in both processes.

## Results

### Altered expression of *miR827* is associated with modified leaf senescence

Our previous analysis revealed that mature *miR827* is highly induced during leaf senescence [37]. Expression analyses of pre*-miR827* resulted in similar kinetics and fold induction, thus verifying enhanced transcript accumulation during early and late stages of natural senescence, as well as during dark-induced senescence of detached leaves (Additional file 1: Figure S1A, C). To determine whether senescence induction by *miR827* is transcriptionally regulated, a ca. 1.3-kb sequence upstream of the transcription start site was cloned in front of the *GFP* reporter gene to construct *miR827Pro:GFP*. *Arabidopsis* plants transformed with this vector were examined for GFP expression. In both natural and artificial senescence of detached leaves, GFP expression was induced during late senescence, with the GFP signal colocalizing with yellowing sections of the leaves (Additional file 1: Figure S1B, D).

**Fig. 1:**
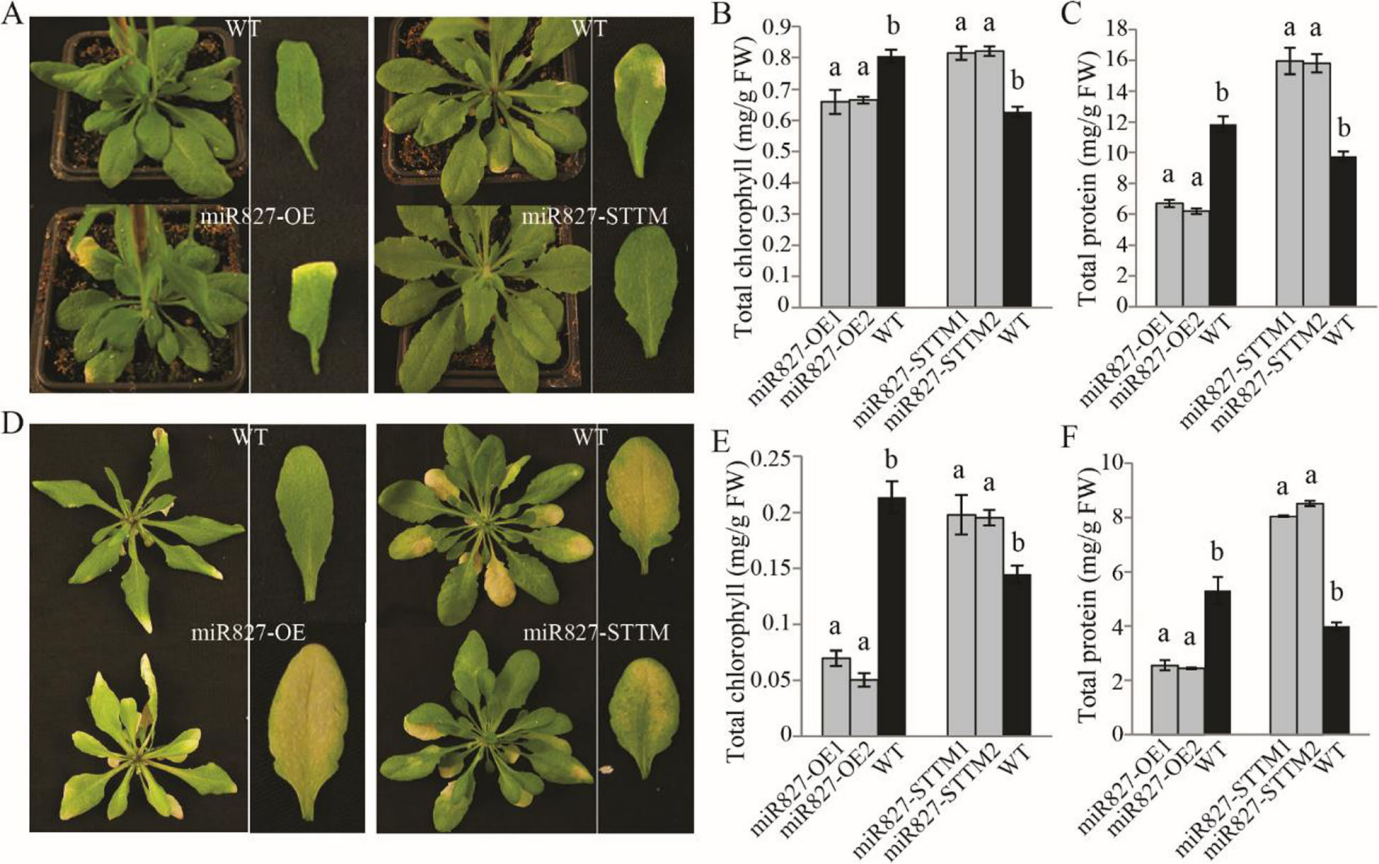
Altered expression of *miR827* in transgenic plants results in modified progression of leaf senescence. **a, d** Enhanced senescence induces leaf yellowing in transgenic plants overexpressing *miR827* (*miR827*–OE) during natural leaf senescence (**a**, left panel) and during artificial dark-induced senescence (**d**, left panel). Retardation of senescence-induced yellowing in *miR827*-silenced transgenic plants (*miR827*–STTM) during natural leaf senescence (**a**, right panel) and during artificial dark-induced senescence (**d**, right panel). **b**, **c** Effects of overexpression or suppression of *miR827* on total chlorophyll (**b**) and protein (**c**) contents during natural leaf senescence. **e**, **f** Effects of overexpression or suppression of *miR827* on total chlorophyll (**e**) and protein (**f**) contents during dark-induced leaf senescence. Three independent transgenic lines were examined. Different letters above the columns indicate significant differences within each compared triplet (*P* < 0.05, Student’s *t* test, ±SD). WT, wild type

To investigate the possible involvement of *miR827* in senescence, pre-*miR827* expression was modified. For overexpression, the *35S* constitutive promoter was cloned in front of the precursor sequences of *miR827* and the constructed vector (Additional file 2: Figure S2A) was used to generate transgenic *miR827*-overexpressing plants in which pre*-miR827* transcript was strongly induced, about 300-fold compared to the wild type (Additional file 2: Figure S2B). Suppression of *miR827* was achieved by employing the target mimicry method (short tandem target mimic—STTM) (Franco-Zorrilla et al., 2007; Yan et al., 2012). The *miR827*–STTM sequence was overexpressed by the *S35* promoter (Additional file 2: Figure S2A), and in the resulting *Arabidopsis* plants, *miR827* transcript level was less than 10% of that measured in the wild type (Additional file 2: Figure S2B). In plants overexpressing pre-*miR827*, accelerated natural senescence of attached leaves was observed as well as acceleration of dark-induced senescence (Fig. 1a, d). Early senescence in the *miR827*-overexpressing lines was indicated by an accelerated decline in chlorophyll and protein contents in same-position leaves (Fig. 1b, c, e, f). In plants suppressed of *miR827* expression retardation of senescence was observed, as indicated by late development of yellowing in the *miR827*–STTM lines (Fig. 1a, d), as well as by a retarded decrease in chlorophyll and protein contents in those lines compared to the wild type during both natural and dark-induced senescence (Fig. 1b, c, e, f).

### Consequences of altered *miR827* expression to gene expression

*NLA* (At1G02860) has been validated as a target of *miR827* in *Arabidopsis* [42, 53], and *PHT5;1/VPT1* (At1G63010) is a suggested target of *miR827* [42]. PHT5;1/VPT1 is essential for Pi homeostasis [54, 55]. A search for additional candidate *miR827* targets using psRNATarget [56] suggested a *GPL* gene (At4G00610), hereafter termed *GPLα*.

**Fig. 2:**
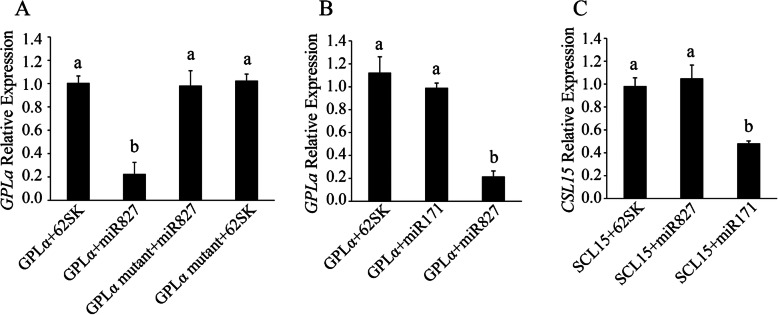
Transcript level of *GPLα* is reduced following transient co-expression with pre-*miR827* in tobacco leaves. **a** *GPLα* or *GPLα* mutated in the putative *miR827*-recognition site was co-expressed with pre-*miR827* or its empty cloning vector (62SK) as a control. Transcript level of *GPLα* was measured by qRT-PCR. **b** *GPLα* was co-expressed with either empty cloning vector control (62SK), pre-*Vv*-*miR171* or pre-*miR827*. **c** *Vv-SCL15* was co-expressed with either pre-*miR827*, pre-*Vv-miR171*, or their empty cloning vector (62SK) as control. Expression was measured by qRT-PCR and presented data are means (*n* = 3). Different letters indicate significant differences (*P* < 0.05, Student’s *t* test, ±SD)

The predicted *miR827*-recognition site in the *GPLα* sequence is shown in Additional file 3: Figure S3. To examine actual cleavage of *GPLα*, 5′ RACE (rapid amplification of cDNA ends) assay was employed. Cleavage of *GPLα* in *Arabidopsis* could not be identified within the predicted target site; it was, however, identified (in 5 out of 13 incidences examined) 10 bases downstream of the 3′-end of the putative recognition site and an additional 8 cleavage sites were spread further downstream (Additional file 3: Figure S3). To further examine the relationship between *miR827* and *GPLα*, we transiently co-expressed both in tobacco leaves using *Agrobacterium* infiltration assay and examined the consequences to *GPLα* transcript level. Co-expression of *GPLα* with pre-*miR827* resulted in a ca. 3-fold decrease in its transcript level compared to co-expression with the empty cloning vector as a control (Fig. 2a). Furthermore, mutations in the predicted *miR827*-recognition site in *GPLα* (Additional file 3: Figure S3) resulted in nullification of the observed *miR827*-mediated decrease in *GPLα* transcript level (Fig. 2a), supporting functional involvement of *miR827* in the regulation of *GPLα*. These mutations by themselves did not affect *GPLα* stability (Fig. 2a). Control experiments revealed the inability of grape *Vv*-*miR171,* which is unrelated to *GPLα*, to affect its transcript levels, whereas it was functional in reducing the transcript level of its verified target *Vv*-*SCL15* (Fig. 2b, c [57]).

**Fig. 3:**
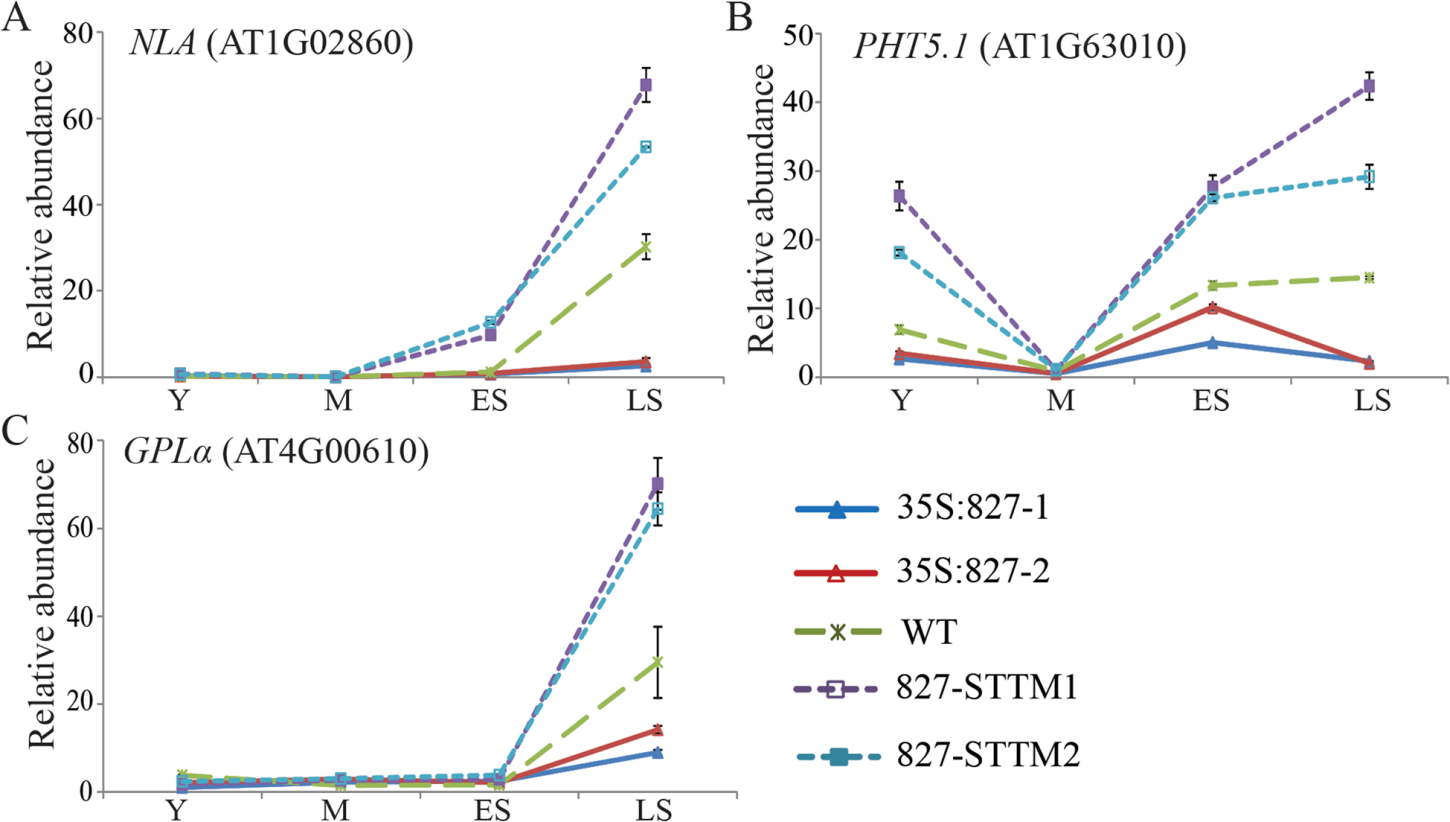
Altered expression of *miR827* affects expression of target genes. **a**–**c** Expression of **a** *NLA* (AT1G02860), **b** *PHT5;1* (PHT5;1/VPT1, AT1G63010), and **c** *GPLα* (AT4G00610) in *miR827*-overexpressing lines (35S:827–1, 2), Col-0 wild type (WT), and *miR827*-silenced lines (827-STTM1, 2) measured by qRT-PCR and representing the mean of three biological repeats. Expression was measured at young (Y), mature (M), early senescence (ES), and late senescence (LS) developmental stages. Error bars correspond to ±SD

Experiments were performed to examine the effects of altered *miR827* expression on its validated target gene *NLA*, as well as on *PHT5;1* and *GPLα* expression. Expression of *NLA*, *PHT5;1*, and *GPLα* was measured during young, mature, early senescence and late senescence stages [37]. In the wild type, all genes were induced toward the late stage of senescence (Fig. 3a–c). *NLA* expression was low at the young, mature, and early senescence stages and then increased in late senescence, as previously reported (Fig. 3a) [37]. *PHT5;1* expression declined at the mature leaf stage compared to that measured at the young leaf stage and then increased again during early and late senescence stages (Fig. 3b). Expression of *GPLα* increased only in late senescence (Fig. 3c). To determine the effects of altering *miR827* expression on these three genes, their expression was measured in transgenic *miR827-*overexpressing and silenced plants. A clear increase in the transcript levels of *NLA*, *PHT5;1*, and *GPLα* was observed in the two *miR827*–STTM lines, whereas their transcript levels were reduced in the two *miR827*-overexpressing lines (Fig. 3a–c).

To further examine the relationship between *miR827* and *GPLα* expression, the latter was characterized in *miR827*-overexpressing and *miR827*-silenced transgenic lines and compared to the expression of two SAGs—*BFN1* (a senescence-induced nuclease [58]) and *SAG12* (a senescence-induced protease [59])—as markers of senescence progression. The expression of *GPLα*, *BFN1*, and *SAG12* was measured at different times during natural leaf senescence and plotted versus chlorophyll content, to enable comparing their expression in the *miR827-*altered lines. Expression of the genes in the wild type was generally correlated and showed an accelerated increase when chlorophyll level was reduced to about 0.5 mg/g fresh weight (Additional file 4: Figure S4). Expression of the three genes was then examined in the transgenic *miR827-*overexpressing *Arabidopsis* in which senescence is enhanced. Expression of *GPLα* was suppressed whereas expression of both *BFN1* and *SAG12* continued to be induced as senescence progressed and chlorophyll levels decreased, suggesting an effect of *miR827* on *GPLα* expression (Additional file 4: Figure S4). On the other hand, expression of *GPLα* in the *miR827*–STTM lines was considerably higher than that of *BFN1* and *SAG12* (Additional file 4: Figure S4), whereas in the wild type, it was lower. The expression of *BFN1* and *SAG12* was induced at a later stage of senescence in the *miR827*–STTM lines, as indicated by a rise in their transcript level when chlorophyll content decreased, relative to that observed in the wild type (Additional file 4: Figure S4).

### Altered *GPLα* expression affects senescence progression

*GPLα* expression increased significantly, mainly during the late stage of natural and dark-induced senescence (Additional file 5: Figure S5A, C). To determine whether senescence induction of *GPLα* is regulated by its promoter, sequences encompassing about 1.3 kb upstream of the transcription start site were cloned in front of the *GFP* coding sequence to construct *GPLαPro:GFP*. Transgenic *Arabidopsis* plants transformed with this vector were examined for GFP expression by following fluorescence. In both natural and artificially (dark)-induced senescence, GFP expression was induced during advanced senescence stages and the GFP signal overlapped with yellowing leaf sections (Additional file 5: Figure S5B, D).

To investigate the involvement of *GPLα* in senescence, its expression was either suppressed or overexpressed in the two *Arabidopsis* accessions: Columbia (Col-0) and Landsberg *erecta* (L*er*). Vectors for generating *GPLα*-overexpressing lines were constructed by activating *GPLα* with the *35S* constitutive promoter (Additional file 6: Figure S6A), resulting in *GPLα–*OE1 and *GPLα–*OE2 (Col-0), and *GPLα–*OE3 and *GPLα–*OE4 (L*er*). *GPLα* expression in the transgenic lines was about 100-fold higher than in the wild type (Additional file 6: Figure S6B, C). Suppression of *GPLα* expression using an RNA interference (RNAi) construct (Additional file 6: Figure S6A) resulted in *GPLα–*SI1 and *GPLα–*SI2 (Col-0), and *GPLα–*SI3 and *GPLα–*SI4 (L*er*), in which *GPLα* expression was suppressed to less than 10% of that measured in the wild type (Additional file 6: Figure S6B, C). In addition, two *Arabidopsis* T-DNA insertional mutants in *GPLα* were used: *ET2099.Ds3.07.28.00.b.544* (Col-0 background) termed *GPLα–*KO1 and *GK-252G05–014573* (L*er* background) termed *GPLα–*KO2 (Additional file 6: Figure S6A). In both of these mutants, expression of *GPLα* was suppressed to less than 10% of that measured in the respective wild types (Additional file 6: Figure S6D).

**Fig. 4:**
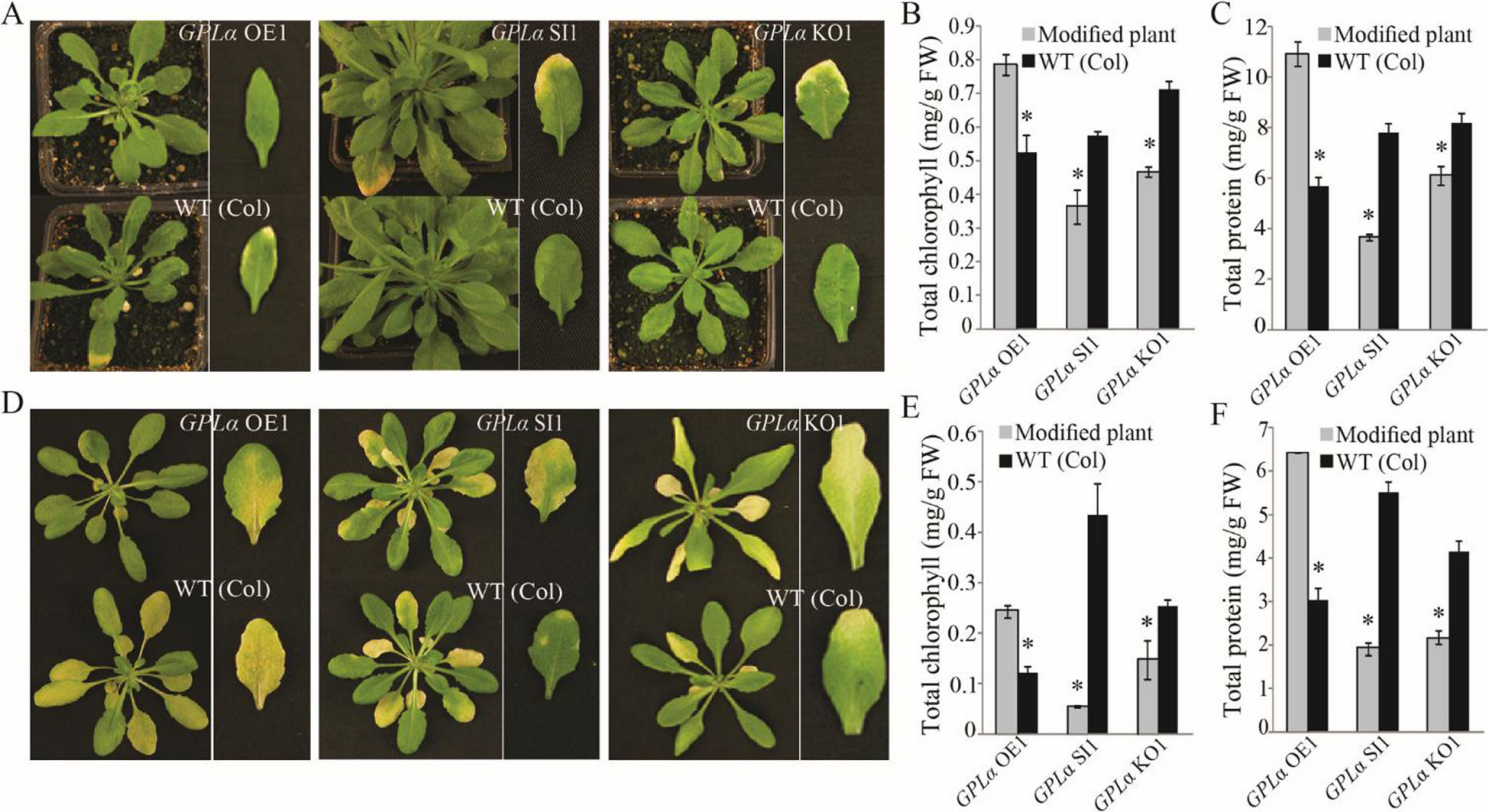
Altered expression of *GPLα* in transgenic plants results in modified progression of leaf senescence. **a**, **d** Inhibited senescence in transgenic plants (Col-0 background) overexpressing *GPLα* (*GPLα* OE) during natural leaf senescence (**a**, left panel) and artificial dark-induced senescence (**d**, left panel). Accelerated senescence in *GPLα-*silenced transgenic plants (*GLPα* SI) during natural leaf senescence (**a**, middle panel) and artificial dark-induced senescence (**d**, middle panel). Accelerated senescence in *GPLα*-mutant plants (*GLPα* KO) during natural leaf senescence (**a**, right panel) and artificial dark-induced senescence (**d**, right panel). **b**, **c** Effects of overexpression or suppression of *GPLα* expression on total chlorophyll (**b**) and protein (**c**) contents during natural leaf senescence. **e**, **f** Effects of overexpression or suppression of *GPLα* expression on total chlorophyll (**e**) and protein (**f**) contents during dark-induced leaf senescence. Three independent transgenic lines were examined. Asterisks indicate significant differences within each compared pair (*P* < 0.05, Student’s *t* test, ±SD). WT (Col), wild type

*GPLα* overexpression resulted in inhibition of both natural (Fig. 4a) and dark-induced (Fig. 4d) senescence in the two independent lines compared to the wild type (Fig. 4a, d). Delayed senescence in the *GPLα*-overexpressing lines was indicated by a retarded decline in both chlorophyll and protein contents in same-position leaves compared to the wild types during natural senescence (Fig. 4b, c) and dark-induced senescence (Fig. 4e, f). Similar retardation of senescence was observed in the L*er* accession (Additional file 7: Figure S7). Suppression of *GPLα* expression resulted in acceleration of both natural (Additional file 7: Figure S7A) and dark-induced (Additional file 7: Figure S7D) senescence. Accordingly, measurements of chlorophyll and protein contents in the *GPLα*-suppressed lines revealed lower levels than those in the wild type during both natural (Additional file 7: Figure S7B, C) and dark-induced (Additional file 7: Figure S7E, F) senescence. Accelerated senescence was apparent when suppression of *GPLα* expression was achieved by either RNAi or T-DNA mutations (Fig. 4). Similar results were obtained for the L*er* accession (Additional file 7: Figure S7).

### Regulation of *miR827* and *GPLα* expression in response to Pi availability and nuclear localization of GPLα

**Fig. 5:**
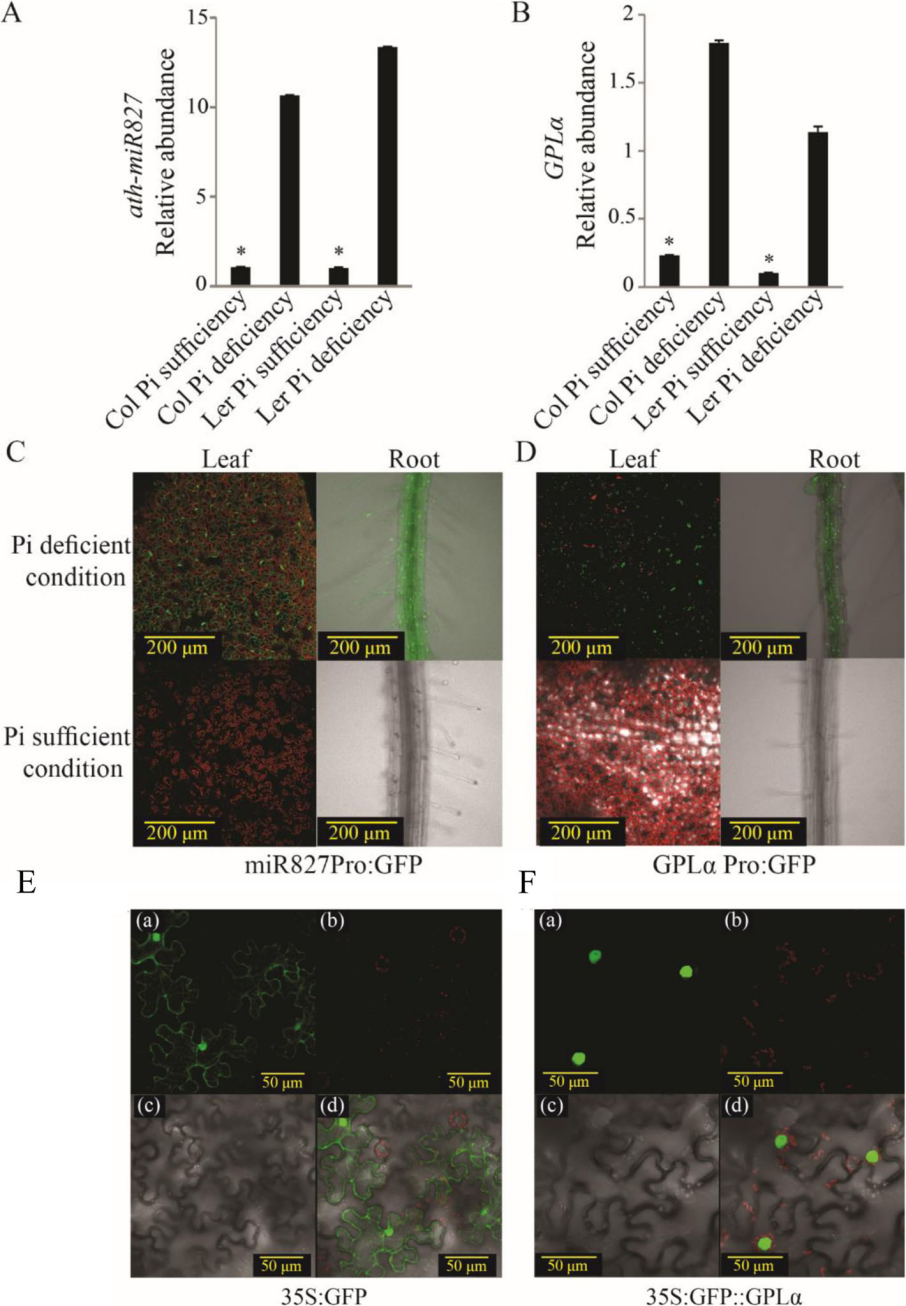
*MiR827* and *GPLα* expression is induced following exposure to Pi-deficient growth conditions and GPLα is localized to *Arabidopsis* cell nuclei. **A** and **B** Measurement of *miR827* (**A**) and *GPLα* (**B**) expression in either Col-0 (Col) or L*er* wild type 10-day-old seedlings following an additional 10 days under Pi-deficient growth conditions. Asterisks indicate significant differences (*P* < 0.05, Student’s *t* test, ±SD). **C** and **D** GFP fluorescence imaging in leaves and roots of transgenic plants (Col-0 background) containing *miR827-*promoter-driven *GFP* (miR827Pro:GFP) (**C**) or *GPLα-*promoter-driven *GFP* (GPLα Pro:GFP) (**D**) under Pi deficiency (upper panels) or optimal Pi (lower panels). Six independent transgenic lines were examined. Representative lines are shown. **E** and **F** *Arabidopsis* wild type (Col-0) leaves were transiently transformed with a vector including the control GFP construct, 35S:GFP (**E**) or a translational fusion between GPLα and GFP, 35S:GFP::GPLα (**F**). GFP protein localization was visualized using confocal microscopy. (**a**) GFP fluorescence; (**b**) chlorophyll autofluorescence; (**c**) bright-field image; (**d**) overlay of **a**, **b**, and **c**

*MiR827* shows increased expression in response to reduced Pi availability [38, 42]. To learn more about the responses of both *GPLα* and *miR827* to Pi limitation, their expression was measured simultaneously in response to transfer from full-nutrient medium to a Pi-deficient one. *Arabidopsis* seedlings (Col-0 and L*er*) were grown for 10 days on half-strength MS medium and then transferred to either full-nutrient or Pi-deficient medium for 10 days and both *miR827* and *GPLα* transcript levels were measured by quantitative (q) RT-PCR. Expression of *miR827*, in both accessions, was found to be induced about 10- to 15-fold under Pi deficiency compared to that measured in the full-nutrient medium, as reported previously [38] (Fig. 5A). *GPLα* expression was also induced, albeit more moderately, under Pi deficiency, increasing to about 4- and 7-fold compared to growth on full-nutrient medium in Col-0 and L*er* accessions, respectively (Fig. 5B).

Expression kinetics of *miR827* and *GPLα* was simultaneously measured following transfer of 10-day-old seedlings from full-nutrient to Pi-deficient medium. Leaf and root tissues were sampled daily for 11 days following the transfer. In leaves, *miR827* expression responded rapidly, in a matter of hours, to the change: it began to increase linearly, reaching about 150-fold its initial expression on day 2 (Additional file 8: Figure S8A). After this striking increase, expression decreased until day 7 to a level that was about 20-fold higher than its initial one; after that, it remained fairly constant (Additional file 8: Figure S8A). Expression of *GPLα*, measured simultaneously in the leaves, changed very mildly, with a tendency to increase until day 4 when it was sharply induced and increased about 25-fold compared to its initial expression (Additional file 8: Figure S8A). After this peak, *GPLα* expression decreased close to its initial expression level.

In the roots, *miR827* expression was induced following exposure to Pi deficiency, similar to its kinetics in the leaves, but to a lower level (Additional file 8: Figure S8B). *MiR827* expression peaked after 2 days of Pi deficiency, to about 8-fold its initial level, followed by a decrease toward day 7, after which it remained low. Expression of *GPLα* remained low initially, increasing only slightly during the first 2 days after exposure to Pi deficiency; this was followed by an increase in expression level to a peak on day 4, at about 12-fold its initial expression (Additional file 8: Figure S8B). This increase occurred concomitantly with the reduction in *miR827* expression level.

To determine whether induction of *miR827* and *GPLα* is regulated by the genes’ upstream promoters, about 1.3 kb upstream of the transcription start site were cloned for each of the genes in front of the *GFP* coding sequence to construct vectors *miR827Pro:GFP* and *GPLαPro:GFP*. Transgenic plants transformed with these vectors were examined for GFP expression by fluorescence imaging. For both promoters, Pi deficiency resulted in induced GFP signal in both leaves and roots (Fig. 5C, D). Under full Pi sufficiency, no GFP signal was detected (Fig. 5C, D).

To localize GPLα protein in the cells, its coding sequence was translationally fused 3′ to the *GFP* coding sequence under the control of the *35S* promoter and the resulting construct was used in an *Arabidopsis* transient-expression assay. Confocal microscopy analyses of the leaves revealed localization of the GFP signal to the nuclei due to the presence of the *GPLα* sequence (Fig. 5F). Localization of GPLα-GFP to the nuclei was verified in transient-expression assay in which GFP signal was observed to overlap with DAPI-stained nuclei (Additional file 9: Figure S9). In control plants, which were infiltrated with *GFP* under the control of the *35S* promoter, GFP signal was present in the cytoplasm as well as in the nuclei, as is characteristic for this protein (Fig. 5E). Nuclear localization of GPLα and the presence of putative nuclear localization sequences were also predicted by the bioinformatics tools ePlant [60] and AtSubP [61], accordingly.

### *MiR827* and *GPLα* are involved in root response to Pi availability

**Fig. 6:**
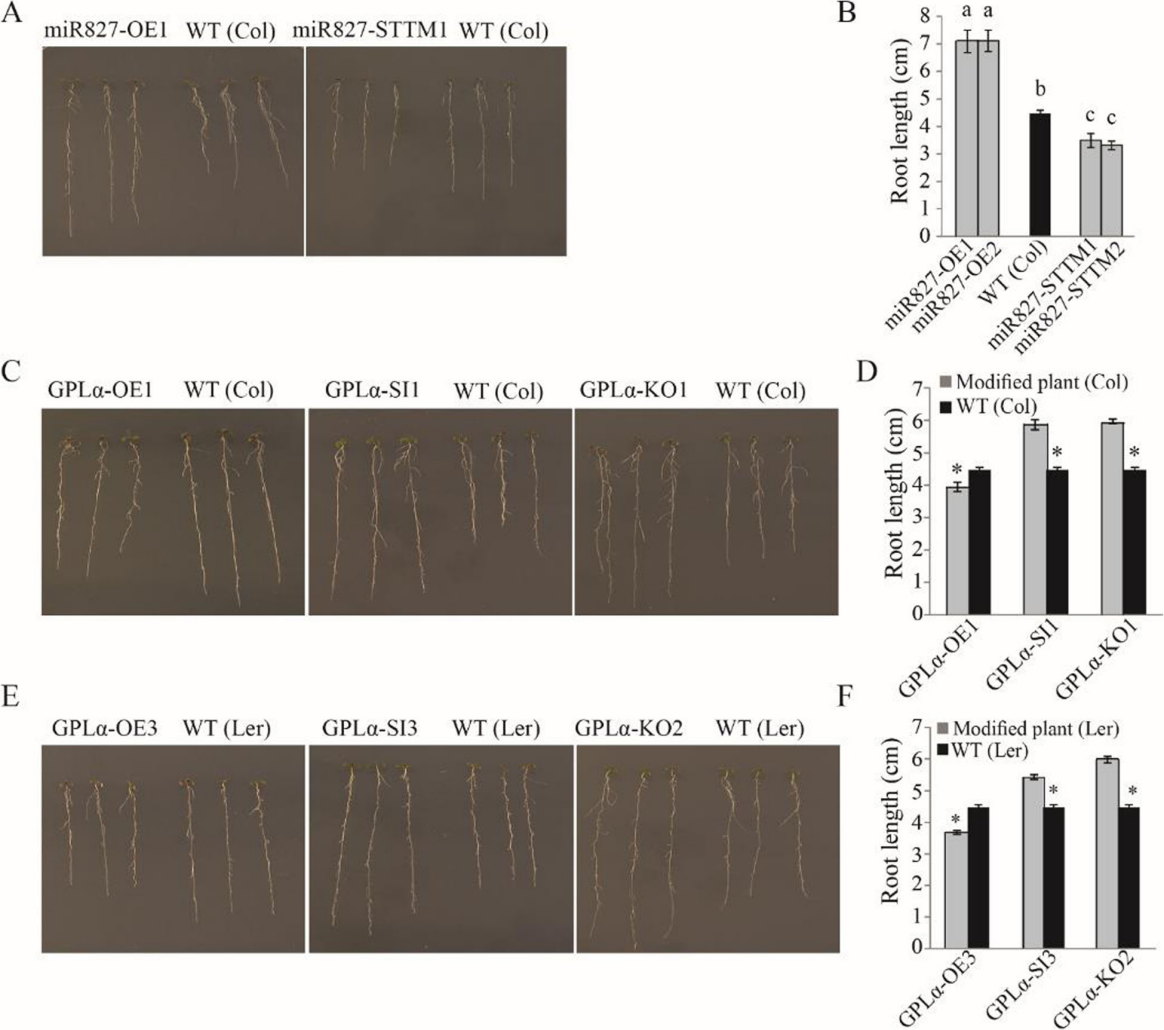
Altered *miR827* or *GPLα* expression affects root-length phenotype under Pi-deficient growth conditions. *Arabidopsis* seedlings were grown on Pi-sufficient medium for 10 days and then transferred to Pi-deficient medium for an additional 7–10 days and root length was measured. **a** Comparison of root-length images from wild type [WT (Col)] and transgenic lines in which *miR827* was overexpressed (*miR827*–OE1 and 2, left image) or suppressed (*miR827*–STTM1 and 2, right image). **b** Quantitative analysis of root-length measurements from WT and *miR827*-overexpressing or suppressed lines in the Col-0 background. **c** Comparison of root-length images from WT (Col) and transgenic lines in which *GPLα* was overexpressed (*GPLα*–OE1; left image), suppressed (GPLα–SI1; middle image), or mutated (GPLα–KO1; right image). **d** Quantitative analysis of root-length measurements from WT and *GPLα*-overexpressing, suppressed, or mutated lines in the Col-0 background. **e** Comparison of root-length images from WT (L*er*) and transgenic lines in which *GPLα* was overexpressed (*GPLα*–OE3; left image), suppressed (*GPLα*–SI3; middle image), or mutated (*GPLα*–KO2; right image). **f** Quantitative analysis of root-length measurements from WT and *GPLα*-overexpressing, suppressed, or mutated lines in the L*er* background. Three independent transgenic lines were examined with measurements of about 30 roots for each of at least three biological repeats. Different letters above the columns indicate significant differences. Asterisks indicate significant differences (*P* < 0.05, Student’s *t* test, ±SD)

Plant developmental responses to Pi deficiency include major alterations in root architecture, such as a reduction in primary-root growth [62, 63]. To determine the possible involvement of *miR827* and *GPLα* in this response, we examined root phenotypes in *Arabidopsis* plants in which expression of these genes was altered. When grown under optimal nutrient conditions, there was no apparent difference in root development between the wild type and either *miR827*-overexpressing/suppressed or *GPLα*-overexpressing/suppressed plants. *Arabidopsis* (Col-0) seedlings were grown on Pi-sufficient medium for 10 days and then transferred to Pi-deficient medium for an additional 7–10 days of growth. Plants overexpressing *miR827* exhibited accelerated growth rate of the primary root, which was thus about 50% longer than that in the wild type (Fig. 6a, b). In *miR827*-suppressed plants grown under Pi deficiency, primary-root elongation was inhibited by about 20% (Fig. 6a, b). In the *Arabidopsis* mutants with modified *GPLα* expression, the opposite effect was observed: under Pi-deficient conditions, primary-root growth was inhibited in the *GPLα*-overexpressing lines, resulting in a low but significant 10% reduction in average primary-root length (Fig. 6c, d). On the other hand, in transgenic plants in which *GPLα* expression was suppressed by either RNAi or T-DNA insertional mutations, primary-root growth was accelerated under Pi deficiency, resulting in an average 20% increase in length (Fig. 6c, d). Similar effects of altered *GPLα* expression on primary-root elongation were observed for the L*er* accession (Fig. 6e, f). The acceleration effect of *GPLα* suppression on root elongation could be reversed by overexpressing *GPLα* in the *GPLα*–KO2 mutant, which resulted in primary-root elongation similar to that measured for the wild type grown under Pi deficiency (Additional file 10: Figure S10).

### GPLα is involved in regulation of *PHT1* expression in response to Pi availability

*MiR827* is known to be involved in Pi homeostasis through the transcript-level regulation of its target gene *NLA* which mediates degradation of the PHT1 family of Pi transporters [39, 64]. We examined the possible involvement of *GPLα* in the regulation of *PHT1* genes as well. *Arabidopsis* seedlings grown for 2 weeks on plates with Pi-sufficient medium were transferred to Pi-deficient medium for 7 days, and gene expression was analyzed. To verify that under our working conditions, Pi-deficiency-induced genes are activated, expression of *PHT1* [51] and *Phosphate1 (PHO1)* [65], encoding for H^+^/Pi cotransporter and the Pi exporter, respectively, was measured following growth on the Pi-deficient medium. Both *PHT1* and *PHO1* are known to be regulated indirectly, at least in part, by *miR827* and *miR399*, respectively [40]. Clear and high induction of transcript levels of both genes was observed (Additional file 11: Figure S11).

**Fig. 7:**
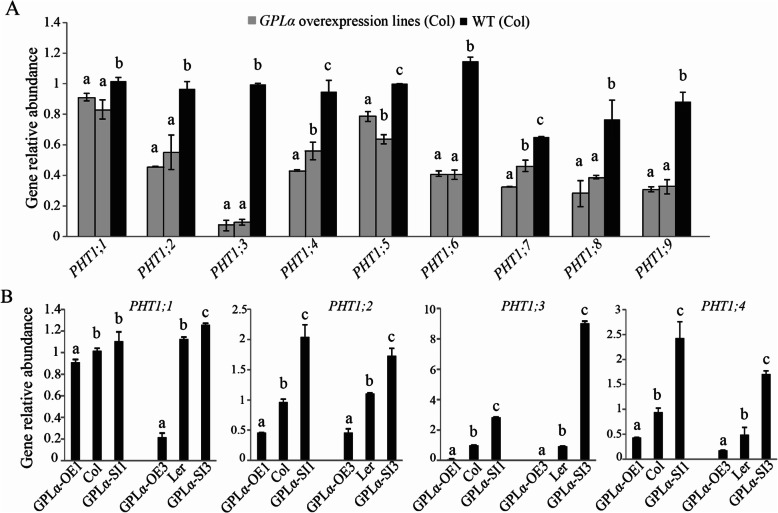
Expression of *PHT1* genes is altered in *GPLα*-modified lines. **a** Expression of the nine *PHT1* family genes in two independent *GPLα*-overexpressing transgenic and wild type [WT (Col)] plants under Pi-deficient growth conditions. **b** Expression of *PHT1;1*, *PHT1;2*, *PHT1;3*, and *PHT1;4* in transgenic lines with modified *GPLα* expression under Pi-deficient conditions including *GPLα*-overexpressing lines *GPLα–*OE1 (Col-0 background) and *GPLα*–OE3 (L*er* background); *GPLα*-suppressed lines *GPLα*–SI1 (Col-0 background) and *GPLα*–SI3 (L*er* background). Expression was measured by qRT-PCR and represents the mean of three biological repeats. Different letters above the columns indicate significant differences within each specific gene triplicate measured in **a** and within each gene triplicate for a given ecotype in **b** (*P* < 0.05, ±SD)

Expression of all nine members of the *PHT1* gene family of *Arabidopsis* [66] was measured in transgenic lines overexpressing *GPLα*. All nine genes had reduced transcript levels in the two independent *GPLα*-overexpressing lines compared to those measured in the wild type following 7 days of growth in Pi-deficient medium (Fig. 7a). For *PHT1;1*, *PHT1;5*, and *PHT1;7*, the reduction was mild, to about 10–30% of that measured in the wild type (Fig. 7a); for *PHT1;3* and *PHT1;6*, the reduction was very strong, 90 and 65% of that found in the wild type, respectively (Fig. 7a). For four of the *PHT1* genes, *PHT1;1* to *PHT1;4*, similar analyses were performed in transgenic *Arabidopsis* lines of both Col-0 and L*er* accessions in which *GPLα* was either overexpressed or suppressed. In both accessions and for all four *PHT1* genes, expression was reduced in the *GPLα*-overexpressing lines and elevated in the *GPLα*-suppressed lines (Fig. 7b). Whereas the effect was mild for *PHT1;1* (Fig. 7b), in *PHT1;3*, both reduction and induction of expression in the *GPLα*-overexpressed and suppressed lines, respectively, were high (Fig. 7b).

To examine whether GPLα is involved in the regulation of other genes known to be induced by Pi deficiency, their expression was examined in the *GPLα*-overexpressing *Arabidopsis* lines. No difference in expression was observed between *GPLα*-overexpressing and wild type lines following exposure to Pi deficiency for any of those genes (Additional file 12: Figure S12).

### Altered *GPLα* expression results in modified Pi levels

**Fig. 8:**
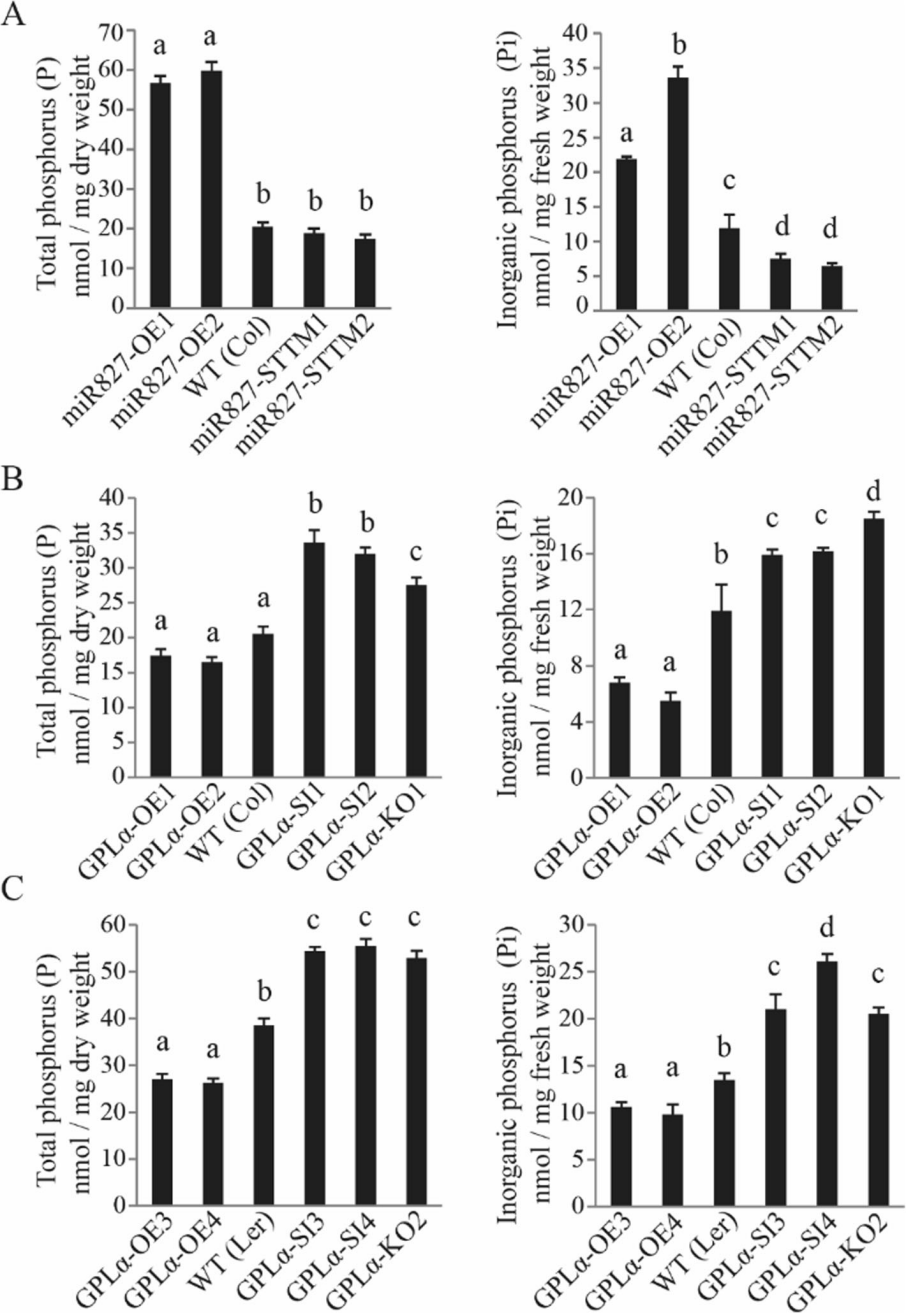
Altered *GPLα* expression results in modified Pi levels in *Arabidopsis* leaves. Total phosphorus (P) and inorganic phosphorus (Pi) content was measured in leaves of *miR827*- and *GPLα-*modified plants grown hydroponically. The Pi and total P contents were measured in 28-day-old plants. **a** Left panel: total P measured in *miR827*-overexpressing lines (*miR827*–OE1 and 2), wild type (Col-0, WT), and *miR827*-suppressed lines (*miR827*–STTM1 and 2). Right panel: Pi measured in these same *miR827*-modified lines. **b** Left panel: total P measured in *GPLα-*overexpressing lines (*GPLα*–OE1 and 2), WT (Col-0), *GPLα*-suppressed lines (*GPLα*–SI1 and 2), and *GPLα-*mutant line (*GPLα*–KO1). Right panel: Pi measured in these same *GPLα*-modified lines. **c** Left panel: total P measured in *GPLα-*overexpressing lines (*GPLα*–OE3 and 4), WT (L*er*), *GPLα*-suppressed lines (*GPLα*–SI3 and 4), and *GPLα-*mutant line (*GPLα*–KO2). Right panel: Pi measured in these same *GPLα*-modified lines. Three independent transgenic lines were examined. Different letters above the columns indicate significant differences (*P* < 0.05, ±SD)

*GPLα* expression was found to be regulated by Pi deficiency, and expression of *PHT1* genes encoding phosphate transporters was altered in transgenic *Arabidopsis* in which *GPLα* expression was modified. Experiments were therefore performed to examine the effects of modified *GPLα* expression on total phosphorus (P) and Pi content. Modulation of *miR827* expression was previously found to affect cellular Pi content, with overexpression of *miR827* resulting in an increase in Pi and P levels [38]. To confirm this effect under our experimental conditions, both Pi and P contents were measured in leaves of *Arabidopsis* lines in which *miR827* was either overexpressed or suppressed and which were grown hydroponically in Pi-sufficient media. Total P content increased to a large extent and more than doubled in two independent transgenic lines in which *miR827* was overexpressed compared to that measured in the wild type (Fig. 8a). On the other hand, suppression of *miR827* did not result in a decrease in P content in the *miR827*-suppressed lines compared to that measured for the wild type plants (Fig. 8a). Pi content increased about 2-fold in the *miR827*-overexpressing lines compared to the wild type, and *miR827* suppression also had an impact on Pi content, resulting in a ca. 30% decrease (Fig. 8a). The effect of *GPLα* expression modification on P and Pi contents was studied in the two *Arabidopsis* accessions, Col-0 and L*er*. In Col-0 *GPLα*-overexpressing lines, total P levels were not significantly different from the wild type although they tended to be lower in the former (Fig. 8b). In the *GPLα*-suppressed lines, including the two independent RNAi lines *GPLα*-SI1 and *GPLα*-SI2 and the T-DNA mutant *GPLα*-KO1, the measured levels of total P were about 30–40% higher than those measured in the wild type (Fig. 8b). Similar measurements of Pi levels revealed about 40–50% reduction in the two *GPLα*-overexpressing lines examined compared to the wild type, whereas in the three independent *GPLα*-suppressed transgenic *Arabidopsis* Col-0 lines, Pi levels were about 25% higher than in the wild type (Fig. 8b). The same analyses were performed in the parallel transgenic *Arabidopsis* L*er* accession in which *GPLα* expression was modified. Total P content was lower in the *GPLα*-overexpressing plants compared to the wild type whereas in the three independent *GPLα*-suppressed plants, P level was higher (Fig. 8c). Pi content in the *GPLα*-suppressed *Arabidopsis* L*er* plants was lower than that in the wild type, but higher than that of the wild type in the transgenic plants with suppressed expression (Fig. 8c).

## Discussion

A marked number of senescence-regulated miRNAs have been reported to be nutrient-responsive [37]. In leaf senescence, nutrient recycling and remobilization to developing sink tissues are of major importance [14, 17]. *MiR408* and *miR827*, which exhibit the strongest induction in expression during leaf senescence, were subjected to further functional analyses. The study of *miR408* revealed its significant involvement in *Arabidopsis* abiotic stress responses [67]. In the current study, we performed a detailed investigation of *miR827*.

### Effect of *miR827*-altered expression on *Arabidopsis* gene expression and leaf senescence

*NLA* has been validated as a target of *miR827* in *Arabidopsis* [42, 53]. NLA, a RING-type ubiquitin E3 ligase, can mediate ubiquitination and degradation of the plasma-membrane-localized PHT1 transporters, thereby negatively regulating their levels and affecting Pi homeostasis [39, 42]. *PHT5;1/VPT1*, suggested to be a target of *miR827* [42, 68], encodes an SPX domain protein that functions in *Arabidopsis* as a vacuolar Pi transporter shown to be essential for Pi homeostasis [54, 55]. In rice, both orthologs of *PHT1;5/VPT1—OsSPX-MFS1* and *OsSPX-MFS2*—were validated as targets of *miR827*, whereas no cleavage of the *OsNLA* transcript, which has a potential *miR827* target, was detected [69]. In this study in *Arabidopsis*, we considered *GPLα* as an additional candidate target of *miR827*. *GPLα* is a member of the *GeBP/GPL* gene family, which encodes unconventional leucine-zipper transcription factors. Different members of this family have been reported to be involved in different developmental and stress responses [70–73].

Expression of *NLA*, *PHT5;1/VPT1*, and *GPLα* was found to be markedly induced when *miR827* expression was also significantly induced (Fig. 3). We previously showed that while full-length *NLA* transcript increases during late senescence, its putative *miR827*-guided cleavage product increases even more strongly [37]. Thus, incoherent senescence regulation is observed for the *NLA* target gene with *miR827*, suggesting that *miR827* moderates the target gene’s expression during senescence rather than acting as an on/off switch.

Clear consequences to the target gene *NLA*, as well as to *PHT5;1/VPT1* and *GPLα* transcript levels, were visualized following manipulation of pre-*miR827* expression in transgenic plants. While the effect on *NLA* transcript level was consistent with it being a target of *miR827* as reported previously [39, 42], the consequences to *GPLα* expression support the notion that it may also be a target of *miR827*.

We examined the consequences of both inhibition and overexpression of pre-*miR827*. Overexpression of a pre-miRNA may not always be fully reflected in equivalently higher levels of the mature miRNA due to possible post-transcriptional processing events [31]. However, our measurements of pre-*miR827* transcript levels during both senescence and Pi starvation were in agreement with the induction of the mature *miR827* [37, 42]. Furthermore, the consequences of pre-*miR827* overexpression for *NLA* level and P/Pi content found in our study (Fig. 3a and Fig. 8) were similar to those reported previously [38, 39, 42].

The effects of pre-*miR827* overexpression and inhibition on *GPLα* transcript level support the possibility that the latter is also a target of *miR827*. Further support stems from the inverse trends in the kinetics of *GPLα* and pre*-miR827* expression under Pi-deficient growth conditions (Additional file 8: Figure S8). A potentially relatively close relationship was also discerned from the differential effect of altered *miR827* expression on *GPLα* expression compared to its effect on other SAGs, including *BFN1*, involved in nuclear DNA degradation during late senescence [58, 74, 75], and *SAG12*, a cysteine protease with a role in N remobilization [27, 76]. The level of *GPLα* expression was clearly more affected than those of *BFN1* and *SAG12* by altered *miR827* expression.

Involvement of *miR827* in the regulation of *GPLα* is also supported by the observed reduction in *GPLα* transcript level following transient co-expression with *miR827*, which was nullified by mutations in the predicted *miR827*-recognition site (Fig. 2). The pairing score of the predicted recognition site in *GPLα* was not the highest; however, it does fulfill the criteria suggested to be required for miRNA targets in *Arabidopsis* [77]. We were able to identify cleavage sites downstream of the predicted recognition site but not in the expected site within *GPLα*. Identification of cleavage sites 3′ to the miRNA recognition site have been suggested to represent rapid and progressive transcript degradation [78, 79].

**Fig. 9:**
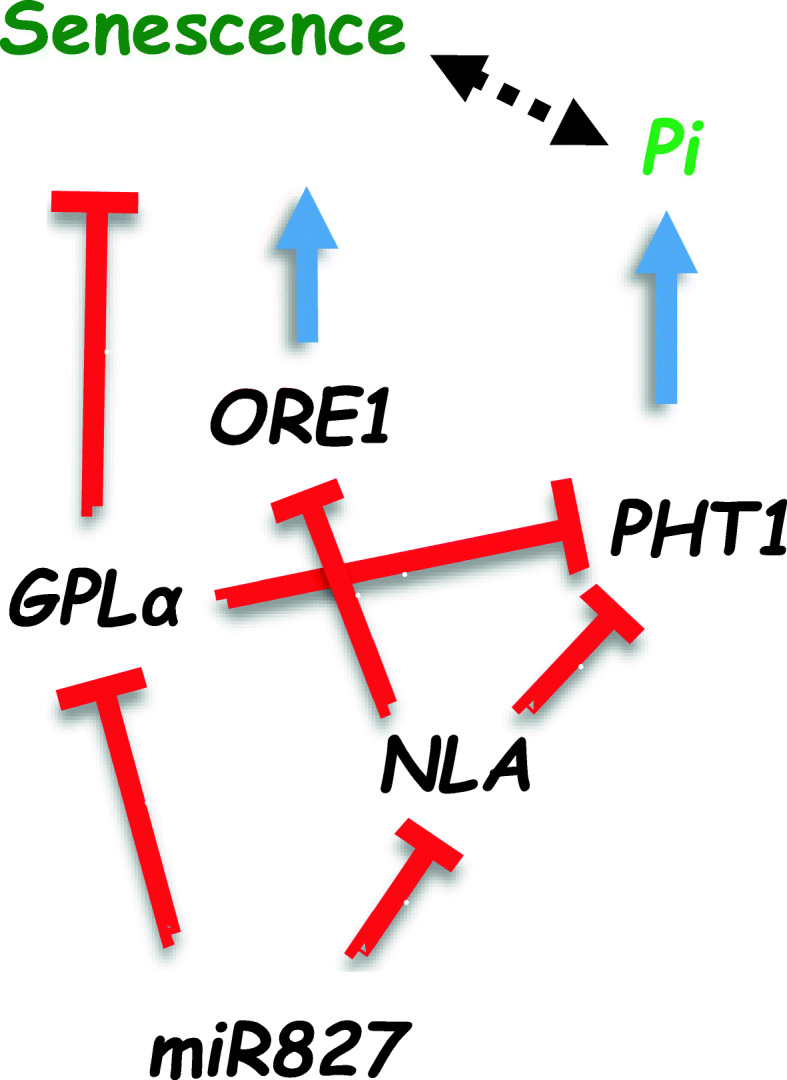
Proposed model for the action of *miR827* and *GPLα*. *MiR827* controls the expression of *PHT1* and Pi levels, by modulating the target *NLA* that negatively controls PHT1 protein level and in parallel, controlling *GPLα* expression, which also negatively controls *PHT1* gene expression. Altered PHT1 levels affect tissue Pi levels. *MiR827* controls the progression of senescence by modulating the target *NLA* that negatively controls protein levels of ORE1, which acts as a positive regulator of senescence, and in parallel modulating the expression of *GPLα*, which negatively controls senescence. *MiR827* induction leads to increased PHT1 levels in parallel to its inducing effect on senescence via its effects on *NLA* and *GPLα.* Negative effect is indicated by red inhibitory signs and positive effect by blue arrows. Double-headed dashed black arrow indicates balanced changes

The effect of altered *miR827* expression on senescence is likely the result of a combined function in regulating the expression of *NLA* and *GPLα*. Mutant plants that were deficient in NLA displayed earlier onset of senescence compared to wild type plants. However, this enhanced-senescence phenotype was only observed under N-limiting conditions and not under optimal growth conditions [80]. The involvement of NLA in senescence induced by N deficiency has been recently revealed [81]. ORE1, known to be key transcription factor regulating age-dependent leaf senescence in *Arabidopsis* [82, 83], was identified as a downstream target of NLA [81]. ORE1 protein stability was found to be controlled by the polyubiquitination–proteasome system involving NLA as an E3 ubiquitin ligase and PHO2/UBC24 as the partner E2 conjugase. It is likely that at least some of the consequences of *miR827* overexpression for senescence acceleration, observed in our study, are mediated via NLA post-translational regulation of ORE1. Elevated *miR827* expression resulted in reduced NLA, which in turn led to elevated ORE1 and acceleration of senescence (Fig. 9). Since in our study, the observed consequence of altered *miR827* expression to senescence was observed under optimal growth conditions, it is suggested that regulation of ORE1 homeostasis by NLA/*miR827* operates not only under N deficiency-induced senescence, but is part of the delicate regulatory mechanism governing fine-tuning control of senescence. In addition, part of *miR827*’s effect on leaf senescence could be attributed to its effect on *GPLα* expression. Induced expression of *miR827* results in inhibited expression of *GPLα*. Since *GPLα* has a negative regulatory effect, senescence is accelerated (Fig. 9).

Altered progress of senescence could result from developmental variability between plants, as well as from different environmental conditions. In this study, comparisons of the different mutant Arabidopsis lines were performed in parallel; thus, the different lines were subjected to the same growth conditions suggesting that the observed modifications in senescence progress are likely resulting from the genetic alterations in *miR827* or *GPLα*. We have not observed any consequences to overall plant development in the different lines investigated, which might have led to alterations in senescence initiation.

### GPLα is involved in regulation of senescence

Functional analysis of *GPLα* indicated its possible involvement in senescence. Under optimal growth conditions, no apparent phenotypes were observed for plants with mutated *GPLα* expression. This fits well with *GPLα*-specific expression in late senescence (Fig. 3). Other *GeBP/GPL* genes, including *GeBP* and *GPL1*, *2*, and *3*, have been shown to be involved in a subset of cytokinin responses [70]. Overexpressing a version of *GPL2* with constitutive transcriptional activation activity exhibited retarded growth, early senescence, and necrotic lesion phenotypes [73]. Phylogeny analysis of the *Arabidopsis GeBP/GPL* family members indicated that *GPLα* is distant from *GeBP* and *GPL1*, *2*, and *3* [71]. Still, GPLα was found to have a repressive effect on senescence resembling the repressive-type function of the other described GeBP/GPL factors.

### Both *miR827* and *GPLα* are involved in regulation of Pi homeostasis in *Arabidopsis*

Previous research has provided ample evidence for *miR827*’s involvement in Pi homeostasis [38, 39]. The observed induction of *GPLα* following exposure to Pi deficiency supports its involvement in Pi homeostasis as well; however, its late induction, 3–4 days after exposure to Pi deficiency, suggests its involvement in a rather late response to the stress compared to *miR827*. In this late P-response stage, when Pi deficiency becomes an extreme stress, a specific set of genes are induced [84] and regulation of Pi homeostasis, with *GPLα* involvement, might change.

Manipulation of *miR827* expression resulted in altered contents of Pi and P (Fig. 8) as reported previously [38]. Interestingly, in both studies, the effect of *miR827* overexpression on P/Pi content was much more significant than that of *miR827* inhibition. This suggests that reducing the level of NLA, which mediates degradation of the PHT1 transporters, has a more prominent role in Pi homeostasis. Accordingly, a mutation in *NLA* results in a more significant effect of increased P/Pi content compared to the effect of overexpressing *NLA*, which results in a mild reduction in P/Pi content in *Arabidopsis* [38].

Altered expression of *GPLα* also had a clear but opposite effect on P/Pi content. Overexpression of *GPLα* resulted in decreased P/Pi levels, whereas suppression did not have a clear effect. Thus, GPLα seems to act as a negative regulator of Pi accumulation, similar to its negative effect on senescence.

Changes in response to Pi deficiency are well known to include a reduction in primary-root growth and an increase in density and length of the lateral roots and root hairs [63, 85, 86]. The consequences of altered expression of either *GPLα* or *miR827* to root development further support the involvement of both genes in Pi homeostasis in *Arabidopsis*. The change in internal Pi level as a result of altered *miR827* expression fits with the observed root-length phenotype. The opposite effect was observed on primary-root length in *GPLα*-transgenic lines (Figs. 6 and 8). These results further support GPLα’s involvement in Pi homeostasis.

NLA directs degradation of the PHT1 transporters [87, 88], and *miR827*’s function in Pi homeostasis is known to be mediated by regulation of *NLA* transcript levels [38, 39]. *GPLα* was found in this study to be involved in the modulation of *PHT1* expression (Fig. 7) with no effect on the expression of a few other examined Pi-starvation-related genes. This inhibitory effect of *GPLα* on *PHT1* expression would explain the reduced levels of P/Pi measured in the *GPLα*-overexpressing lines.

Plant Pi transporters are known to be under complex regulation, by different transcription factors, such as MYB-, WRKY-, or BHLH-type involved in transcriptional regulation of *PHT* [64, 89]. The central and important regulatory role of *GPLα* is suggested by its effect on the expression of all *PHT1* genes. *GPLα* has a negative regulatory effect on *PHT1* gene expression as well as on P/Pi content in the plant. Similar negative regulation on Pi-stress-induced responses in plants has been suggested for MYB62 [90].

Thus, a negative regulatory function of GPLα is suggested for both senescence and Pi-related responses. Other members of the *GeBP/GPL* gene family have been previously suggested to function as negative regulators: *GeBP* was suggested to act as a repressor of leaf cell fate [71], and *GPL1*, *GPL2*, and *GPL3* were shown to play a repressive role in the determination of final organ size and cell expansion [73]. These *GPL* genes have also been suggested to play a role in the cytokinin pathway mediated by their repressive function on *Arabidopsis* response regulators [70]. A recent investigation of *GPL4* suggested its involvement in the roots’ response to toxic metals [72]. Thus, although these different members of the *GeBP/GPL* gene family seem to function in very different aspects of plant development and stress response, their mode of action seems to include a negative regulatory aspect, as observed in our study for the effect of *GPLα* on senescence and Pi homeostasis.

## Conclusions

Our current study suggests a regulatory function for both *miR827* and *GPLα* in Pi homeostasis as well as in senescence, as suggested in the model presented in Fig. 9. Induced expression of *miR827* results in reduced expression of *NLA* which is known to negatively regulate *ORE1*, thus resulting in accelerated senescence. At the same time, induced *miR827* results in decreased *GPLα* expression, also associated with accelerated senescence. Thus, the function of *miR827* for senescence induction could be mediated in parallel by its effects on either NLA or GPLα. Interestingly, a similar parallel function of *miR827* is proposed to affect Pi homeostasis via its negative regulatory effect on either *NLA* or *GPLα*, both having a negative regulatory effect on *PHT1* genes. Thus, *miR827* activation enables increased Pi transport due to its effect on increasing PHT1 levels via its effects on both *NLA* and *GPLα*. This suggested regulatory circuit involving *miR827*, *GPLα*, *NLA*, and *ORE1* could be part of the complex regulatory mechanism of senescence. Many studies support the view that the onset and progression of senescence are highly controlled, to enable efficient recycling of nutrients present in the leaves. Pi is an important nutrient that requires appropriate regulatory and functional components for its recycling, including the functioning of phosphate transporters such as PHT1s to enable export of the nutrient from the senescing tissue. The functions of *miR827*, *NLA*, *GPLα*, *ORE1*, and *PHT1* and the interactions between them could be part of the mechanism controlling the balance between senescence progression, especially in its advanced stage, and recycling and transport of Pi for efficient nutrient recycling, which is the essence of senescence.

## Methods

### Plant growth conditions

*Arabidopsis thaliana* ecotypes Columbia (Col-0) and Landsberg *erecta* (L*er*) were the wild type plants used. Two *GPLα* T-DNA insertional lines (ET2099.DS3.07.28.00.B.544, GK-252G05-014573) were ordered from the ABRC stock center. Seeds were germinated on soil or half-strength MS medium following 2 days of vernalization at 4 °C. Plants were grown at 22 °C under a 16 h/8 h light/dark cycle. The complete medium contained the following: 2 mM Ca(NO_3_)_2_, 2.5 mM KH_2_PO_4_, 1 mM MgSO_4_, 5 mM KNO_3_, 7 μM H_3_BO_3_, 50 μM FeSO_4_, 50 μM Na-EDTA, 14 μM MnCl_2_, 0.7 μM ZnSO_4_, 0.2 mM Na_2_MoO_4_, and 50 nM CuSO_4_. To control Pi levels, KH_2_PO_4_ was replaced with K_2_SO_4_ [38, 39]. To examine the effects of Pi deficiency, 10-day-old seedlings were transferred to normal or Pi-deficient plates.

For experiments in which senescence was evaluated, either naturally or artificially induced senescing *Arabidopsis* plants were used. Single plants were grown in potting soil mixture Green 7611 (Green Ltd., http://www.evenari.co.il) in 7 cm × 7 cm × 8 cm containers at 22 °C under a light/dark cycle of 16/8 h (long days). No fertilizer was added. For natural senescence, when initial yellowing tissue, indicative of senescence, was observed in one of the lines examined in a given experiment, leaves 5 and 6 were harvested from multiple plants, pooled, and frozen for further analyses. This usually occurred at about 35–45 days under our growth conditions which are similar to previously described kinetics of natural leaf senescence in Arabidopsis [22]. For artificially induced senescence experiments, *Arabidopsis* plants were grown as described above, until central stems were initiated. Intact plants were removed from the soil, their roots were excised, and they were placed in the dark, in containers fitted with inlet and outlet ports. The containers were sealed and connected to a flow-through system of air with a flow rate maintained at 50 mL/min, bubbled through sterile water to maintain humidity in the container. Once leaves 5 and 6, in one of the lines, initiated senescence, they were harvested from multiple plants, pooled, and frozen for further experiments.

### Vector construction and plant transformation

Full-length pre-miRNA of *miR827* (miRBase accession no. MI0005383, TAIR accession no. AT3G59884) and *GPLα* (TAIR accession no. AT4G00610) were cloned from *Arabidopsis*. PCR amplification primers were designed by the software Primer Premier 6, and amplified PCR fragments were cloned into the *Xho*I–*Xba*I (*miR827*) or *Nco*I–*Xba*I (*GPLα*) restriction sites of the plant expression vector *pFGC5941* (GenBank accession no. AY310901), resulting in vectors *pFGC5941-p35S-miR827* and *pFGC5941-p35S-GPL*, respectively. STTM strategy was used to silence *miR827* [91]. The STTM sequence was synthesized (GENEWIZ) and cloned into the *pFGC5941* vector restriction sites *Nco*I–*Xba*I, resulting in vector *pFGC5941-p35S-827STTM*. For the *GPLα*-silencing vector, a segment of the *GPLα* cDNA sequence was PCR amplified and cloned as two inverted repeats into the restriction sites *Xho*I–*Nco*I and *Xba*I–*Bam*HI of vector *pFGC5941*, resulting in RNAi vector *pFGC5941-p35S-GPL-SI*.

Genomic *Arabidopsis* DNA, including the 1325-bp 5′ sequence of the *miR827* promoter or the 2030-bp sequence of *GPLα* up to ATG, were cloned into restriction sites S*al*I–N*ot*I, of the pORE R4 vector (ordered from TAIR) [92], resulting in vector *pFGC-5941-p827-GFP* and *pFGC-5941-pGPL-GFP*, respectively. For subcellular localization analysis, *GPLα* cDNA, including the coding sequences, was cloned upstream or downstream of the *GFP* cDNA coding sequences to generate either N- or C-terminal translational fusions of *GPLα* and *GFP* coding sequences (GPLα–GFP, GFP–GPLα) using the Gateway cloning system (Invitrogen, pDONR/SD/D TOPO and pK7FWG2.0, pK7FWF2.0 vectors). Chimeric genes were cloned under the *35S* promoter. All of the primers are listed in Additional file 13: Table S1.

For construction of vectors for transient expression, ath-pre-*miR827* and the full-length *GPLα* cDNA coding sequences were used, as well as those of grape *Vvi-miR171d* and *Vv-SCL15*. The predicted *miR827* target site in *GPLα* was mutated at the coding sequence nucleotide positions 792 and 795, replacing T with C (Additional file 3: Figure S3). The pre-*miR827* or pre-*Vvi-miR171d* were cloned into the vector pGreen II-62-SK (Youbio, China), while *GPLα* or *SCL15* coding sequences were cloned into the vector pGreen II-0800-miRNA (Youbio). All cDNAs were cloned under the *35S* promoter.

Transformation of *Arabidopsis* plants was performed by *Agrobacterium*-mediated floral dip method [93]. Homozygous lines were established and T3 or T4 lines were used for the experiments. Experiments designed to examine the effects of *miR827* on *GPLα* were performed by transient co-expression of the relevant expression vectors following *Agrobacterium* infiltration of *Nicotiana benthamiana* leaves. The expression vectors were cotransformed with the helper pSoup19 into *Agrobacterium* strain GV3101 used for tobacco-infiltration experiments. The bacteria were suspended in infiltration medium (10 mM MgCl_2_, 10 mM MES-KOH, pH 5.2, 0.1 mM acetosyringone) at OD_600_ = 1, and incubated for 4–5 h at room temperature. The bacterial suspension was injected into the leaves with a syringe. Zones of infiltrated tissues were harvested 4 days after injection for RNA isolation.

### Protein and chlorophyll content measurements

*Arabidopsis* leaf was extracted with 150 μL extraction buffer (50 mM Tris-HCl pH 7.5, 0.1% w/v SDS, 10 mM EDTA, and 1 mM PMSF) by crushing in a 2010 Geno Grinder (SPEX Sample Prep). For the chlorophyll assay, 170 μL H_2_O was added to 30 μL of the extract, followed by vortexing and extraction with 800 μL acetone. Following 10 min centrifugation at maximal speed, the supernatant was used to measure absorbance for quantification of chlorophyll a + b [94]. For protein concentration measurements, the remaining extract was centrifuged at 18,000*g* at room temperature for 10 min and the clear supernatant was used to measure protein content with a protein assay kit (BioRad).

### Quantitative RT-PCR

Total RNA was isolated using the Spectrum Plant Total RNA kit (Sigma-Aldrich) and RNA was reverse-transcribed with the Verso cDNA Synthesis kit (Thermo Scientific) using gene-specific primers or oligo(dT). The qRT-PCR was performed with a StepOne™ Real-Time PCR System (Applied Biosystems) using Fast SYBR® Green Master Mix (Applied Biosystems) and gene-specific primers (Additional file 13: Table S2). The qRT-PCR primers were designed with Primer Express 3.0 software (Applied Biosystems). Expression data were analyzed by CT (cycle threshold) value [95, 96]. All experiments were carried out with non-template controls and in three biological repeats.

### Transient expression in *Arabidopsis* and confocal microscopy fluorescence observations

Transient expression in *Arabidopsis* was performed for localization experiments using the vectors harboring the GPLα-GFP and GFP-GPLα translational fusions, as well as the GFP included in the pFGC5941 vector as a control. Vectors were transformed into *Agrobacterium tumefaciens* strain EHA105 by the heat-shock transformation method. *Agrobacterium* leaf infiltration was performed as described previously [97]. Confocal microscopy observation and image acquisition were carried out with an Olympus IX 81 inverted confocal laser scanning microscope (FLUOVIEW 500) equipped with a 488-nm argon-ion laser and 60X 1.0 NA Plan Apo water-immersion objective. GFP was excited by 488 nm light and the emission was collected through a BA 515–525 filter; a BA 660 IF emission filter was used to collect chlorophyll autofluorescence. Confocal optical sections were obtained at 0.5-μm increments.

### Fluorescence imaging

GFP fluorescence imaging of leaves of transgenic plants was performed using the IVIS Lumina II imaging system (PerkinElmer). To measure GFP fluorescence, appropriate filters were used to obtain 430 nm excitation and 530 nm emission. The conditions used for imaging included 12.5 cm field of view for sample, 1.2 lens aperture f/stop, and medium pixel binning (CCD resolution) (to balance pixel size and sensitivity). Acquisition time was 3–5 s. Data capture and analysis were performed using IVIS Lumina II Living Image® Software.

### Quantification of total P and soluble Pi

Total P and soluble Pi contents were analyzed as described previously [98, 99] with minor modifications. Total P quantification was performed using leaf tissue dried at 80 °C for 24 h. A 100-mg sample of dry leaf material was dissolved in 2 mL sulfuric acid and digested in a heat block at 250 °C for 1 h. After cooling to room temperature, 300 μL H_2_O_2_ was added to the solution; the solution was heated to 250 °C for 10 min and then cooled to room temperature. These steps were repeated until the solution was clear. The solution was diluted 50-fold in water and used to measure P concentration. For soluble Pi measurement, fresh tissue was crushed in liquid N and homogenized with 1% glacial acetic acid. Then, 50 μL sample was mixed with 250 μL water and 700 μL assay buffer (600 μL solution of 0.42% w/v NH_4_MoO_4_ and 0.86 N H_2_SO_4_, 100 μL 10% ascorbic acid) and incubated at 42 °C for 20 min. Pi content was assayed by measuring absorbance at 820 nm.

### Accession numbers

Sequence data from this article can be found in The Arabidopsis Information Resource (TAIR) or the National Center for Biotechnology Information (NCBI) under the following Arabidopsis Genome Initiative (AGI) locus identifiers: *miR827* (At3g59884), *GPLα* (At4g00610), *NLA* (At1g02860), *SPX* (At1g63010), *SAG12* (At5g45890), *BFN1* (At1g11190), *PHO1* (At3g23430), *PHO2* (At2g33770), *PHT1;1* (At5g43350), *PHT1;2* (At5g43370), *PHT1;3* (At5g43360), *PHT1;4* (At2g38940), *PHT1;5* (At2g32830), *PHT1;6* (At5g43340), *PHT1;7* (At3g54700), *PHT1;8* (At1g20860), *PHT1;9* (At1g76430), *PHT2* (At3g26570), *PHF1* (At3g52190), *AtIPK1* (At5g42810), *AtPAP12* (At2g27190), *AtPAP26* (At5g34850), *ZAT6* (At5g04340), *ACTIN* (AtT3g18780), and *UBC* (At5g25760).

## Supplementary Information

**Additional file 1: Fig. S1.** Expression of pre-*miR827* is induced during natural and dark-induced leaf senescence and is regulated by upstream sequences. A and C Expression of pre-*miR827* during natural senescence (A) and artificial dark-induced senescence (C) was measured by qRT-PCR in young (Y), mature (M), early senescence (ES) and late senescence (LS) leaves, and in late-stage dark-induced senescence (DIS). Different letters and asterisk indicate significant difference (*P* < 0.05, Student’s *t* test, ±SD). B and D GFP fluorescence measured in leaves of transgenic plants containing *miR827*-promoter-driven *GFP* (miR827Pro:GFP) during natural senescence (B) or dark-induced senescence (D). Six independent transgenic lines were examined and representative lines are shown. WT, wild type.

**Additional file 2: Fig. S2.** Vector constructs and effects of genetic manipulations of pre-*miR827* expression level on transgenic plants. A Upper map: transformation vector constructed for overexpression of *miR827*. The precursor of *miR827* is regulated by the constitutive *35S* promoter. Lower map: transformation vector constructed for silencing of *miR827* using a target mimic method (STTM) to silence *miR827* activated constitutively by control of the *35S* promoter. Construction details are described in Methods. B Expression levels of pre-*miR827* in two independent transgenic lines overexpressing *miR827* (35S:827-OE1, 2), the wild type [WT (Col)], and two independent *miR827*-silenced lines (35S:827-STTM1, 2). Expression was measured by qRT-PCR and represents the mean of three biological repeats; values were normalized to WT levels. Asterisk indicates significant difference from WT (*P* < 0.05, Student’s *t* test, ±SD).

**Additional file 3: Fig. S3.** Predicted *miR827*-recognition site in the *GPLα* sequence. Upper row, sequence of *GPLα* transcript from nucleotides 778–825. Sequence identified as putative target for *miR827* is labeled in red and the extent of base-pairing to the miR827 sequence shown in the lower row is presented. Two U positions changed to C in the mutant version of GPLα are shown in blue above the recognition site. Cleavage-site position experimentally identified by 5′ RACE analysis for 5 out of 13 incidences examined is indicated by a vertical arrow. Another 8 sites were spread among 5 additional locations in the 100-bp region downstream of the putative recognition site.

**Additional file 4: Fig. S4.** Senescence-associated expression of *GPLα* is altered in transgenic plants with modified *miR827* expression. Expression levels of *GPLα*, *SAG12* and *BFN1* were measured by qRT-PCR in leaves of *miR827*-overexpressing (*miR827*–OE1), wild-type (WT) (Col-0) and *miR827-*silenced (*miR827*–STTM1) lines at different senescence stages. Relative abundance of the transcripts of the three genes was plotted against chlorophyll content, representing senescence stage. Three independent transgenic lines were examined. Error bars correspond to ± SD. FW, fresh weight.

**Additional file 5: Fig. S5.** Expression of *GPLα* is induced during natural and dark-induced leaf senescence and is regulated by upstream sequences. A and C Expression of *GPLα* during natural senescence (A) and artificial dark-induced senescence (C) was measured using qRT-PCR in young (Y), mature (M), early senescence (ES), and late senescence (LS) leaves, and in late dark-induced senescence (DIS). Different letters and asterisk indicate significant difference (*P* < 0.05, Student’s *t* test, ±SD). B and D GFP fluorescence measured in leaves of transgenic plants containing *GPLα-*promoter-driven *GFP* (GPLαPro:GFP) during natural senescence (B) or dark-induced senescence (D). Three independent transgenic lines were examined and representative lines are shown. WT, wild type.

**Additional file 6: Fig. S6.** Vector constructs and effects of genetic manipulations of *GPLα* expression level on transgenic plants. A Upper map: transformation vector constructed for overexpression of *GPLα*. Full-length *GPLα* coding sequence was cloned and regulated by the constitutive *35S* promoter. Middle panel: map of the constructed transformation vector for silencing *GPLα*. The RNAi gene constructed to silence *GPLα* was regulated by the constitutive *35S* promoter. Construction details are described in Methods. Lower map: genomic sites of T-DNA insertions for the two independent *GPLα*-mutant lines—*ET2099.Ds3.07.28.00.b.544* (*GPLα*–KO1) and *GK-252G05–014573* (*GPLα*–KO2). B–D Expression levels of *GPLα* in transgenic plants overexpressing *GPLα* (*GPLα* OE1, 2), or with silenced *GPLα* (*GPLα* SI1, 2), and in the wild type [WT (Col)] (B), and in transgenic plants *GPLα* OE3, 4, *GPLα* SI3, 4, and WT (L*er*) (C). Expression levels of *GPLα* in the *GPLα* mutants in the two ecotypes (*GPLα* KO1 in Col-0, *GPLα* KO2 in L*er*) (D). Expression was measured by qRT-PCR and represents the mean of three biological repeats. Asterisk indicates significant difference from WT (*P* < 0.05, Student’s *t* test, ±SD).

**Additional file 7: Fig. S7.** Altered expression of *GPLα* in transgenic plants results in modified progression of leaf senescence. A and D Inhibited senescence in transgenic plants (L*er* background) overexpressing *GPLα* (*GPLα* OE3) during natural leaf senescence (A, left panel) and artificial dark-induced senescence (D, left panel). Accelerated senescence in *GPLα-*silenced transgenic plants (*GPLα* SI3) during natural leaf senescence (A, middle panel) and artificial dark-induced senescence (D, middle panel). Accelerated senescence in *GPLα-*mutant plants (*GPLα* KO2) during natural leaf senescence (A, right panel) and artificial dark-induced senescence (D, right panel). WT (L*er*), wild-type L*er* accession. B and C Effects of *GPLα* overexpression or suppression on total chlorophyll (B) or protein (C) contents during natural leaf senescence. E and F Effects of overexpression or suppression of *GPLα* expression on total chlorophyll (E) and protein (F) contents during dark-induced leaf senescence. Three independent transgenic lines were examined. Asterisks indicate significant differences within each compared pair (*P* < 0.05, Student’s *t* test, ±SD).

**Additional file 8: Fig. S8.** Induction of pre-*miR827*, *GPLα* and *PHT1* expression following exposure to Pi-deficient growth conditions. A and B Wild-type (Col-0) seedlings (10 days old) were transferred to Pi-deficient medium and expression was measured simultaneously in leaves (A) and roots (B) at different times after transfer. The primers used to measure *PHT1* gene expression enabled the measurement of the total transcript of a few different members of the *PHT1* gene family. The primers (Table S2) fully matched *PHT1;1* and *PHT1;2* and likely recognized a few additional *PHT1* members with some mismatches in the primer sequences. Error bars indicate ±SD.

**Additional file 9: Fig. S9.** GPLα-GFP is localized to the nuclei. Leaves were transiently transformed with a vector including a translational fusion between GPLα and GFP, 35S:GFP::GPLα. GFP protein localization was visualized using confocal microscopy. (a) GFP fluorescence; (b) DAPI nuclei staining dye fluorescence; (c) bright-field image; (d) overlay of a, b, and c.

**Additional file 10: Fig. S10.** Measured root length for the mutant line *GPLα–*KO2 in which *GPLα* was overexpressed [*GPLα–*OE (*GPLα–*KO2)]. The mutant line *GPLα*–KO2 (L*er* background) was transformed for overexpression of *GPLα* and the consequences for root length following exposure to growth under Pi deficiency were examined in three independent transgenic lines. Different letters above the columns indicate significant differences (*P* < 0.05, ±SD). WT, wild type.

**Additional file 11: Fig. S11.** Expression of *PHT1* and *PHO1* is induced under Pi-deficient conditions. Expression of *PHT1* (A) and *PHO1* (B) was measured in both Col-0 (Col MS) and L*er* (Ler MS) wild-type accessions following transfer of seedlings to Pi-deficient growth conditions for 7 days (Col MS-Pi and Ler MS-Pi, respectively). Expression was measured by qRT-PCR and represents the mean of three biological repeats. Asterisks indicate significant difference (*P* < 0.05, Student’s t-test, ±SD). The primers used to measure *PHT1* expression were as described for Additional file 8.

**Additional file 12: Fig. S12.** Expression analysis of Pi-deficiency-responsive genes in *GPLα*-overexpressing lines. Expression was measured by qRT-PCR and represents the mean of three biological repeats. Different letters above the columns indicate significant differences (*P* < 0.05, ±SD). WT, wild type.

**Additional file 13: Table S1.** Sequences of primers used in gene cloning and vector construction. **Table S2.** Primers used for qRT-PCR analysis of gene expression.

## Data Availability

All data generated or analyzed during this study are included in this published article and its supplementary information files.

## Abbreviations

GPLα: GLABRA1 enhancer-binding protein (GeBP)-like
SAG: Senescence-associated gene
miRNA: MicroRNA
NLA: Nitrogen limitation adaptation
PHT1: Phosphate transporter 1
Col-0: Columbia
L*er*: Landsberg *erecta*
