# Supplementary figures and images for: Downregulation of GeBP-like α factor by MiR827 suggests their involvement in senescence and phosphate homeostasis
Source: BMC Biol. 2021 May 3;19:90. doi: 10.1186/s12915-021-01015-2 (PMC8091714; doi:10.1186/s12915-021-01015-2)

## Slide 1
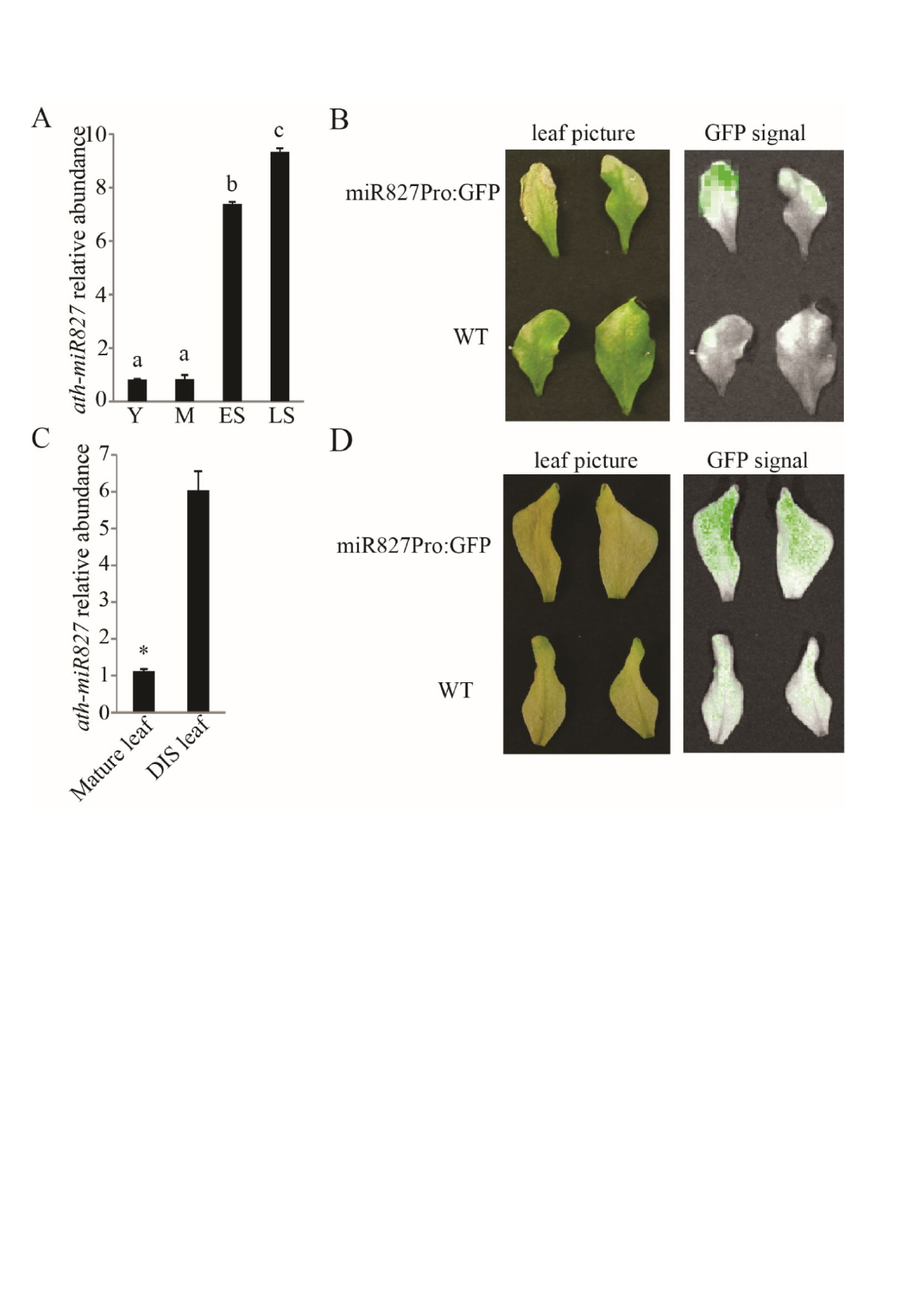

Supplement: Supplementary file 1 — Additional file 1: Fig. S1. Expression of pre-miR827 is induced during natural and dark-induced leaf senescence and is regulated by upstream sequences. A and C Expression of pre-miR827 during natural senescence (A) and artificial dark-induced senescence (C) was measured by qRT-PCR in young (Y), mature (M), early senescence (ES) and late senescence (LS) leaves, and in late-stage dark-induced senescence (DIS). Different letters and asterisk indicate significant difference (P < 0.05, Student’s t test, ±SD). B and D GFP fluorescence measured in leaves of transgenic plants containing miR827-promoter-driven GFP (miR827Pro:GFP) during natural senescence (B) or dark-induced senescence (D). Six independent transgenic lines were examined and representative lines are shown. WT, wild type. [file 12915_2021_1015_MOESM1_ESM.pptx]

## Slide 1
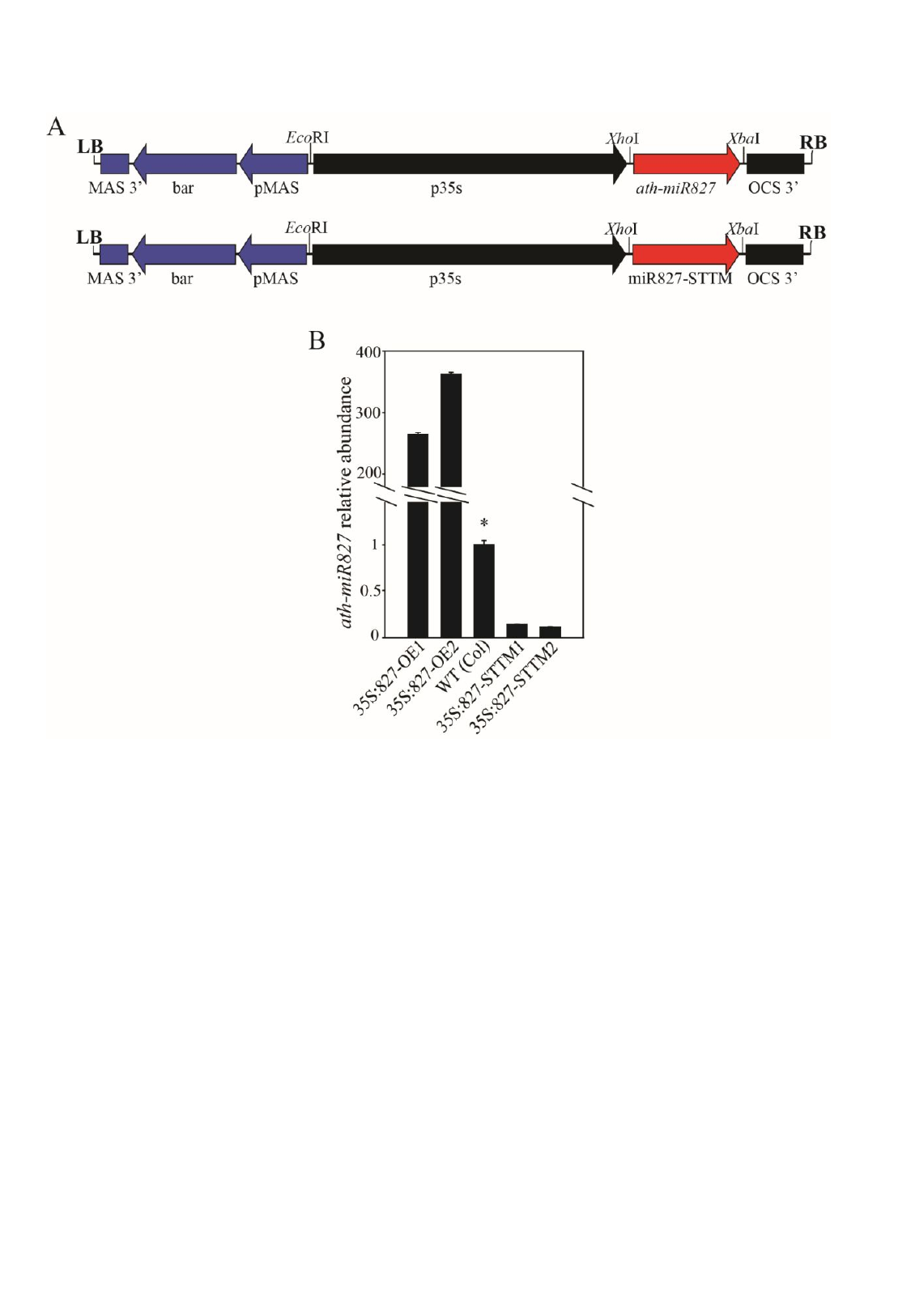

Supplement: Supplementary file 2 — Additional file 2: Fig. S2. Vector constructs and effects of genetic manipulations of pre-miR827 expression level on transgenic plants. A Upper map: transformation vector constructed for overexpression of miR827. The precursor of miR827 is regulated by the constitutive 35S promoter. Lower map: transformation vector constructed for silencing of miR827 using a target mimic method (STTM) to silence miR827 activated constitutively by control of the 35S promoter. Construction details are described in Methods. B Expression levels of pre-miR827 in two independent transgenic lines overexpressing miR827 (35S:827-OE1, 2), the wild type [WT (Col)], and two independent miR827-silenced lines (35S:827-STTM1, 2). Expression was measured by qRT-PCR and represents the mean of three biological repeats; values were normalized to WT levels. Asterisk indicates significant difference from WT (P < 0.05, Student’s t test, ±SD). [file 12915_2021_1015_MOESM2_ESM.pptx]

## Slide 1
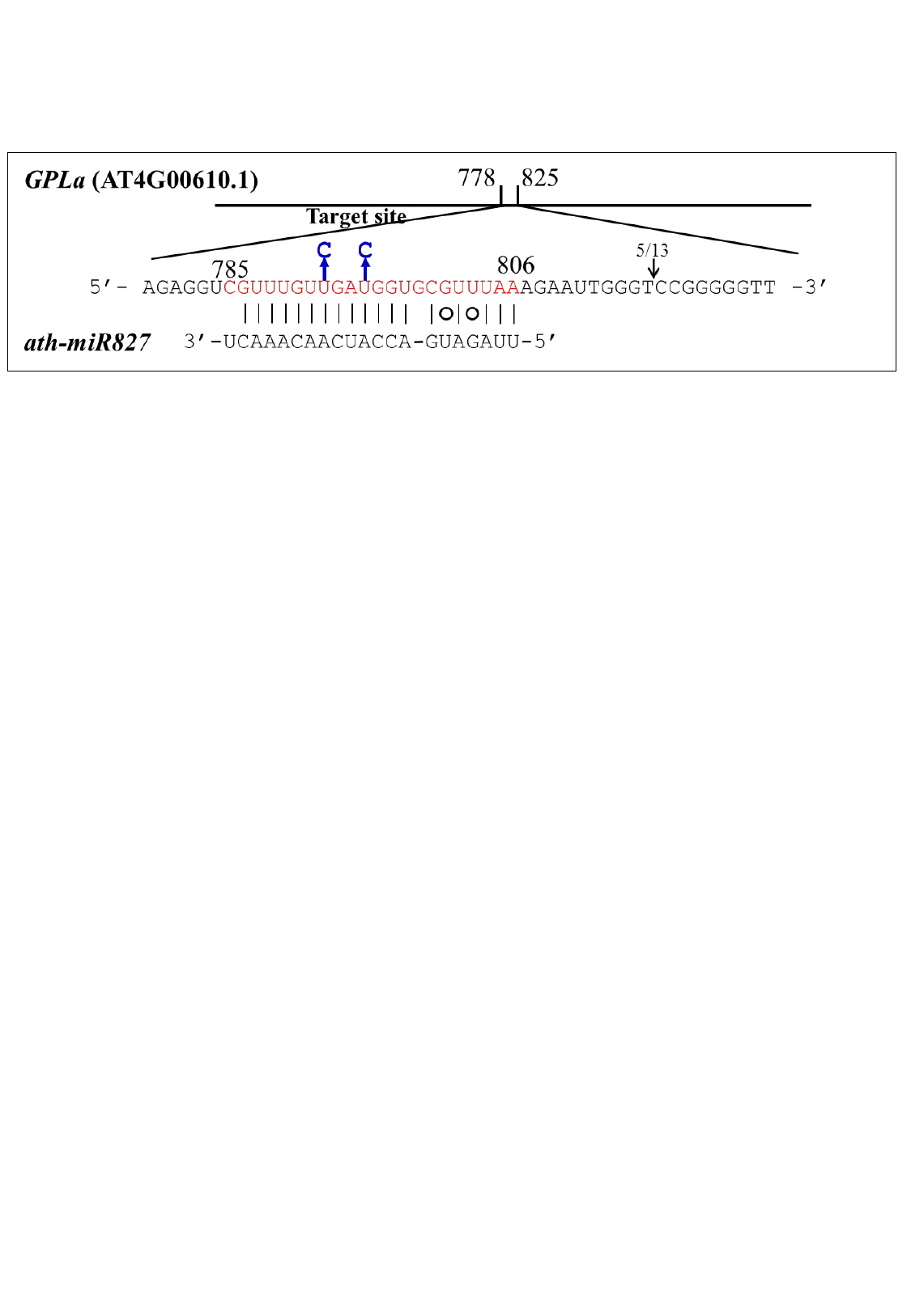

Supplement: Supplementary file 3 — Additional file 3: Fig. S3. Predicted miR827-recognition site in the GPLα sequence. Upper row, sequence of GPLα transcript from nucleotides 778–825. Sequence identified as putative target for miR827 is labeled in red and the extent of base-pairing to the miR827 sequence shown in the lower row is presented. Two U positions changed to C in the mutant version of GPLα are shown in blue above the recognition site. Cleavage-site position experimentally identified by 5′ RACE analysis for 5 out of 13 incidences examined is indicated by a vertical arrow. Another 8 sites were spread among 5 additional locations in the 100-bp region downstream of the putative recognition site. [file 12915_2021_1015_MOESM3_ESM.pptx]

## Slide 1
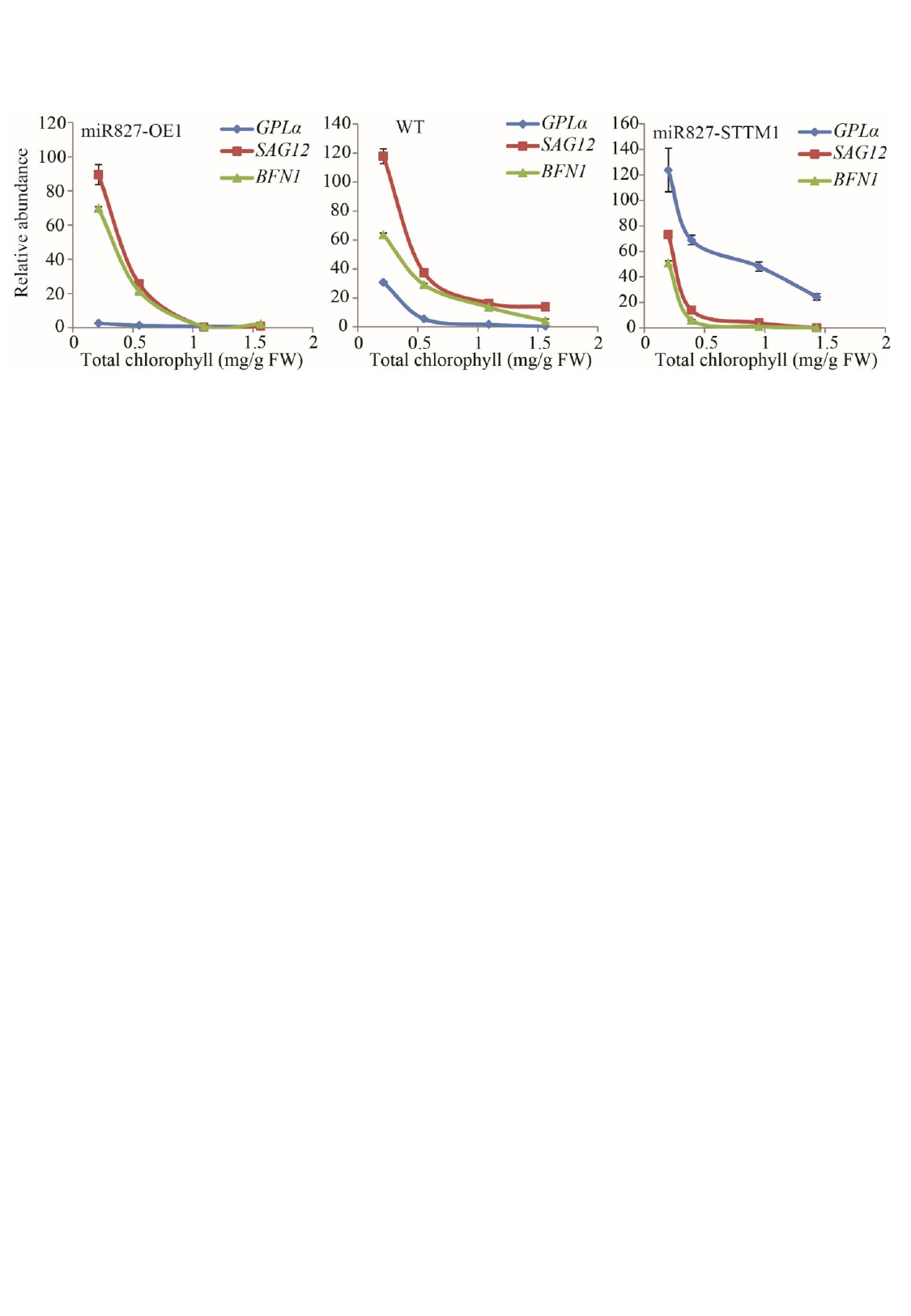

Supplement: Supplementary file 4 — Additional file 4: Fig. S4. Senescence-associated expression of GPLα is altered in transgenic plants with modified miR827 expression. Expression levels of GPLα, SAG12 and BFN1 were measured by qRT-PCR in leaves of miR827-overexpressing (miR827–OE1), wild-type (WT) (Col-0) and miR827-silenced (miR827–STTM1) lines at different senescence stages. Relative abundance of the transcripts of the three genes was plotted against chlorophyll content, representing senescence stage. Three independent transgenic lines were examined. Error bars correspond to ± SD. FW, fresh weight. [file 12915_2021_1015_MOESM4_ESM.pptx]

## Slide 1
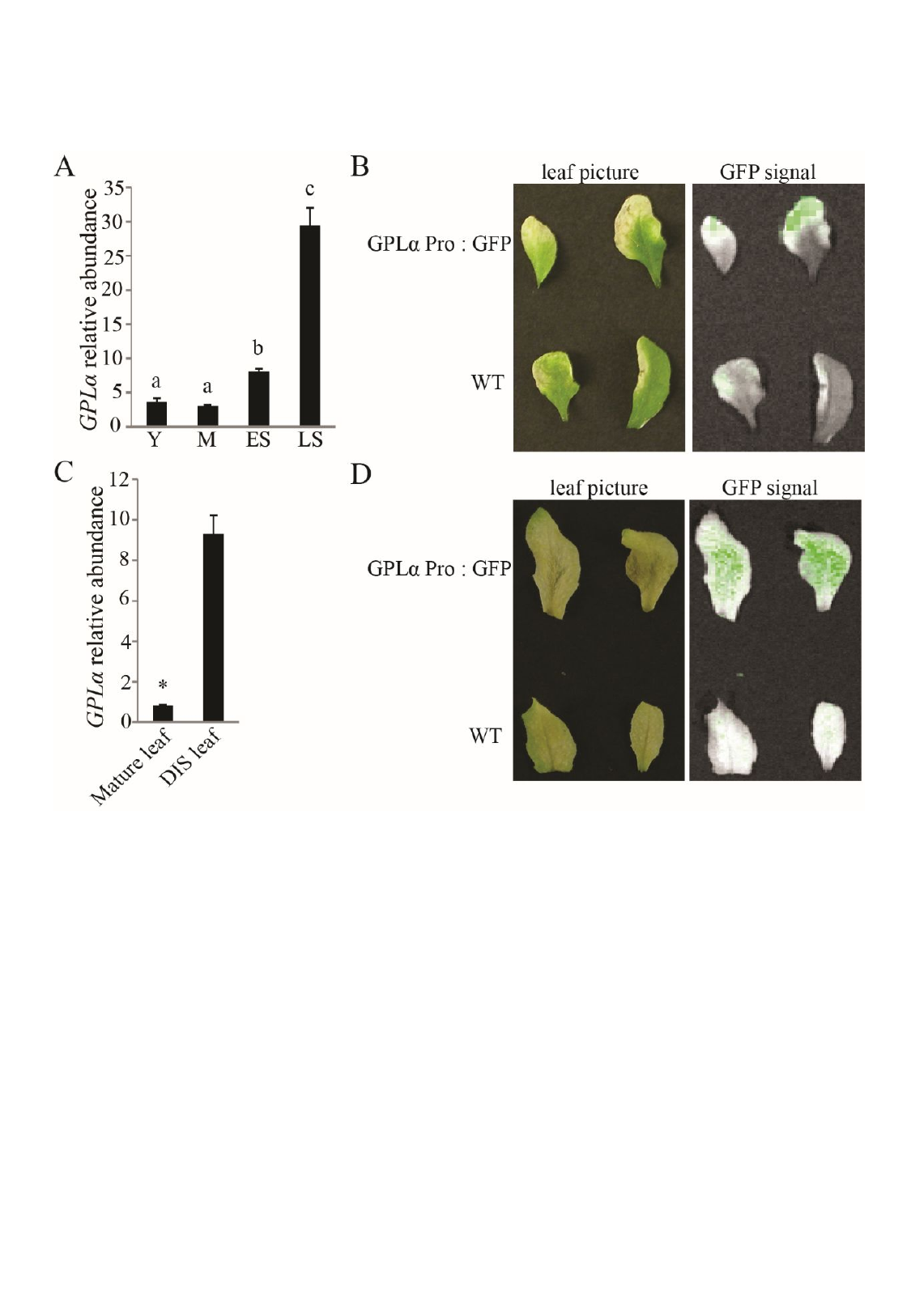

Supplement: Supplementary file 5 — Additional file 5: Fig. S5. Expression of GPLα is induced during natural and dark-induced leaf senescence and is regulated by upstream sequences. A and C Expression of GPLα during natural senescence (A) and artificial dark-induced senescence (C) was measured using qRT-PCR in young (Y), mature (M), early senescence (ES), and late senescence (LS) leaves, and in late dark-induced senescence (DIS). Different letters and asterisk indicate significant difference (P < 0.05, Student’s t test, ±SD). B and D GFP fluorescence measured in leaves of transgenic plants containing GPLα-promoter-driven GFP (GPLαPro:GFP) during natural senescence (B) or dark-induced senescence (D). Three independent transgenic lines were examined and representative lines are shown. WT, wild type. [file 12915_2021_1015_MOESM5_ESM.pptx]

## Slide 1
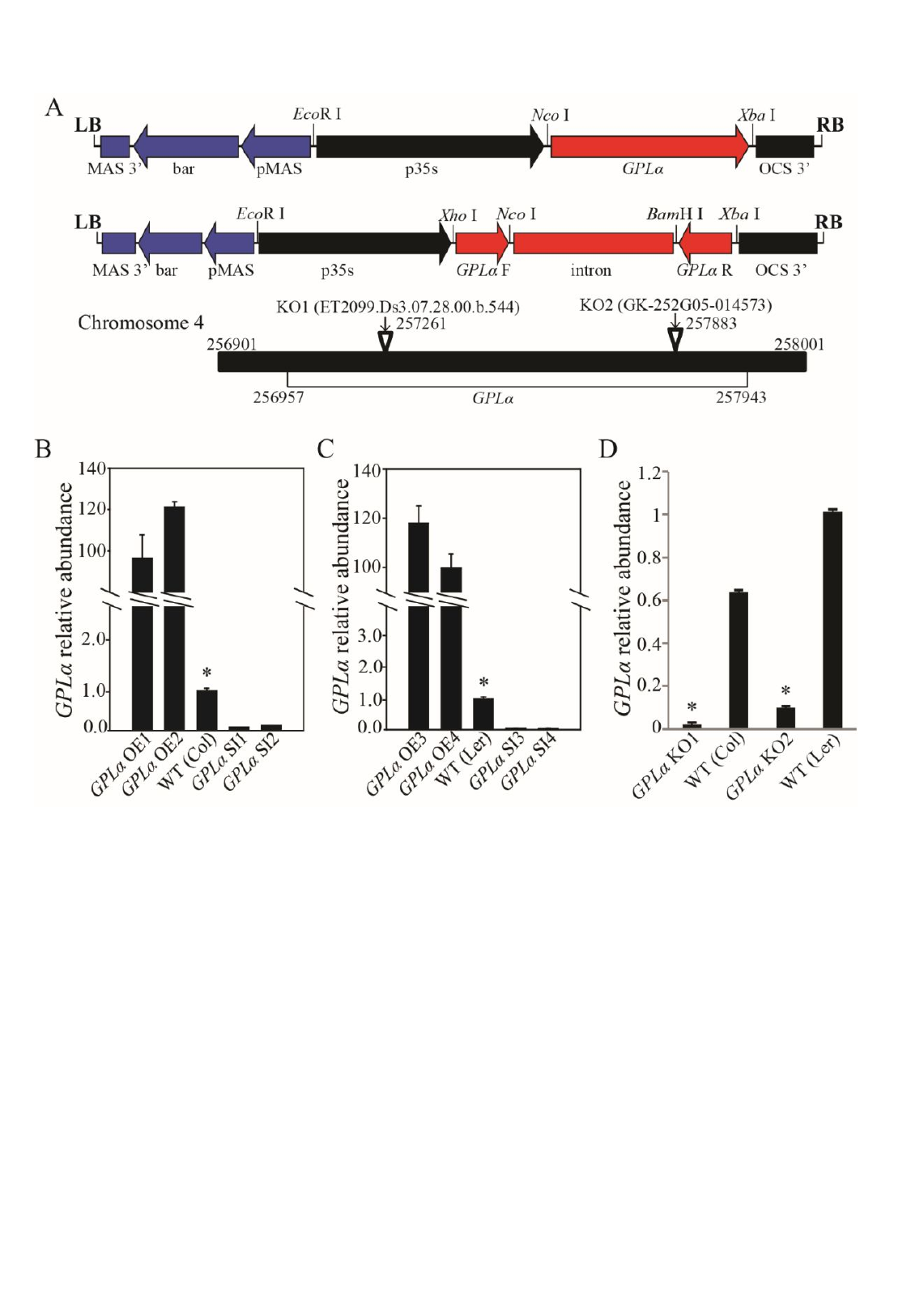

Supplement: Supplementary file 6 — Additional file 6: Fig. S6. Vector constructs and effects of genetic manipulations of GPLα expression level on transgenic plants. A Upper map: transformation vector constructed for overexpression of GPLα. Full-length GPLα coding sequence was cloned and regulated by the constitutive 35S promoter. Middle panel: map of the constructed transformation vector for silencing GPLα. The RNAi gene constructed to silence GPLα was regulated by the constitutive 35S promoter. Construction details are described in Methods. Lower map: genomic sites of T-DNA insertions for the two independent GPLα-mutant lines—ET2099.Ds3.07.28.00.b.544 (GPLα–KO1) and GK-252G05–014573 (GPLα–KO2). B–D Expression levels of GPLα in transgenic plants overexpressing GPLα (GPLα OE1, 2), or with silenced GPLα (GPLα SI1, 2), and in the wild type [WT (Col)] (B), and in transgenic plants GPLα OE3, 4, GPLα SI3, 4, and WT (Ler) (C). Expression levels of GPLα in the GPLα mutants in the two ecotypes (GPLα KO1 in Col-0, GPLα KO2 in Ler) (D). Expression was measured by qRT-PCR and represents the mean of three biological repeats. Asterisk indicates significant difference from WT (P < 0.05, Student’s t test, ±SD). [file 12915_2021_1015_MOESM6_ESM.pptx]

## Slide 1
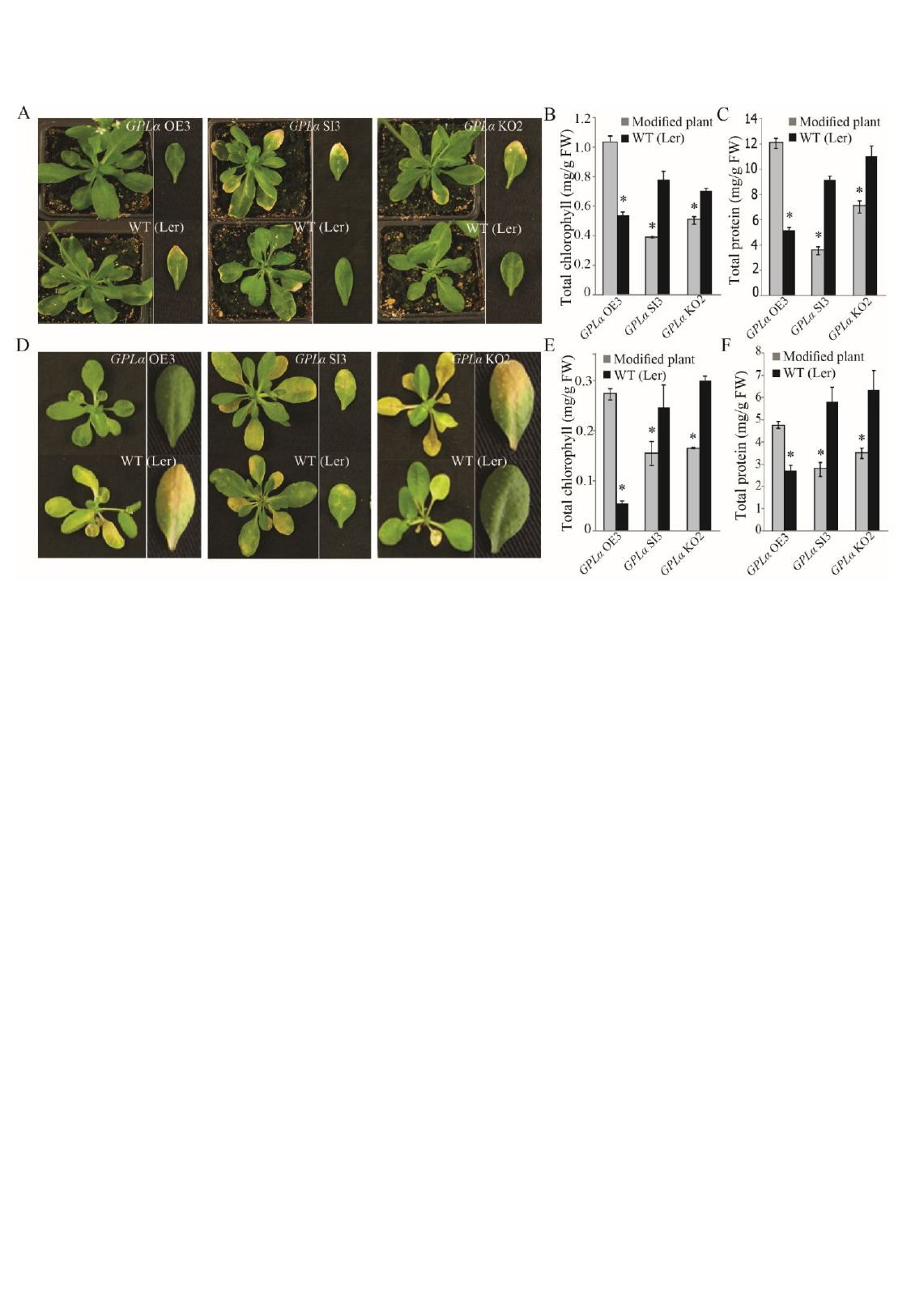

Supplement: Supplementary file 7 — Additional file 7: Fig. S7. Altered expression of GPLα in transgenic plants results in modified progression of leaf senescence. A and D Inhibited senescence in transgenic plants (Ler background) overexpressing GPLα (GPLα OE3) during natural leaf senescence (A, left panel) and artificial dark-induced senescence (D, left panel). Accelerated senescence in GPLα-silenced transgenic plants (GPLα SI3) during natural leaf senescence (A, middle panel) and artificial dark-induced senescence (D, middle panel). Accelerated senescence in GPLα-mutant plants (GPLα KO2) during natural leaf senescence (A, right panel) and artificial dark-induced senescence (D, right panel). WT (Ler), wild-type Ler accession. B and C Effects of GPLα overexpression or suppression on total chlorophyll (B) or protein (C) contents during natural leaf senescence. E and F Effects of overexpression or suppression of GPLα expression on total chlorophyll (E) and protein (F) contents during dark-induced leaf senescence. Three independent transgenic lines were examined. Asterisks indicate significant differences within each compared pair (P < 0.05, Student’s t test, ±SD). [file 12915_2021_1015_MOESM7_ESM.pptx]

## Slide 1
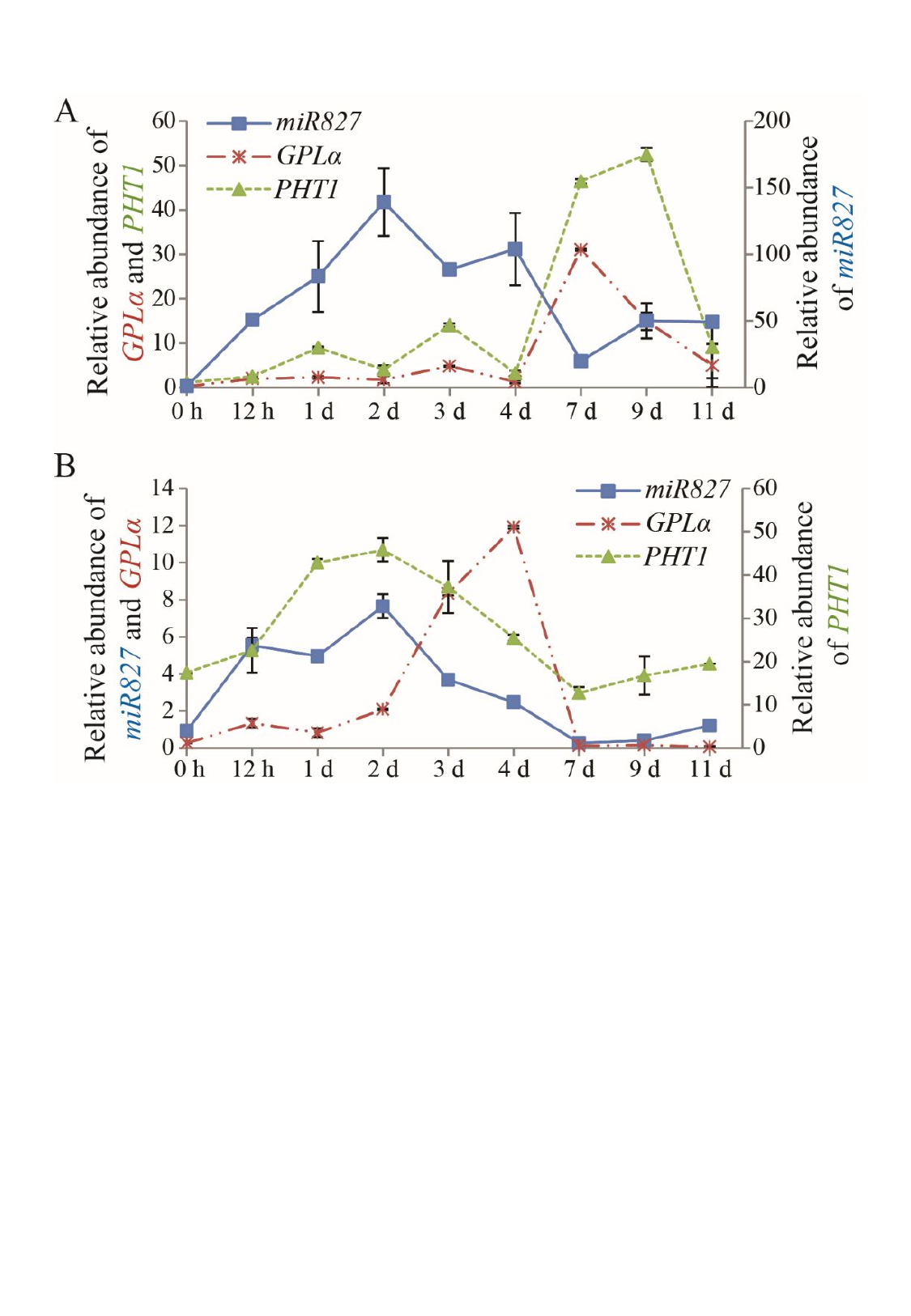

Supplement: Supplementary file 8 — Additional file 8: Fig. S8. Induction of pre-miR827, GPLα and PHT1 expression following exposure to Pi-deficient growth conditions. A and B Wild-type (Col-0) seedlings (10 days old) were transferred to Pi-deficient medium and expression was measured simultaneously in leaves (A) and roots (B) at different times after transfer. The primers used to measure PHT1 gene expression enabled the measurement of the total transcript of a few different members of the PHT1 gene family. The primers (Table S2) fully matched PHT1;1 and PHT1;2 and likely recognized a few additional PHT1 members with some mismatches in the primer sequences. Error bars indicate ±SD. [file 12915_2021_1015_MOESM8_ESM.pptx]

## Slide 1
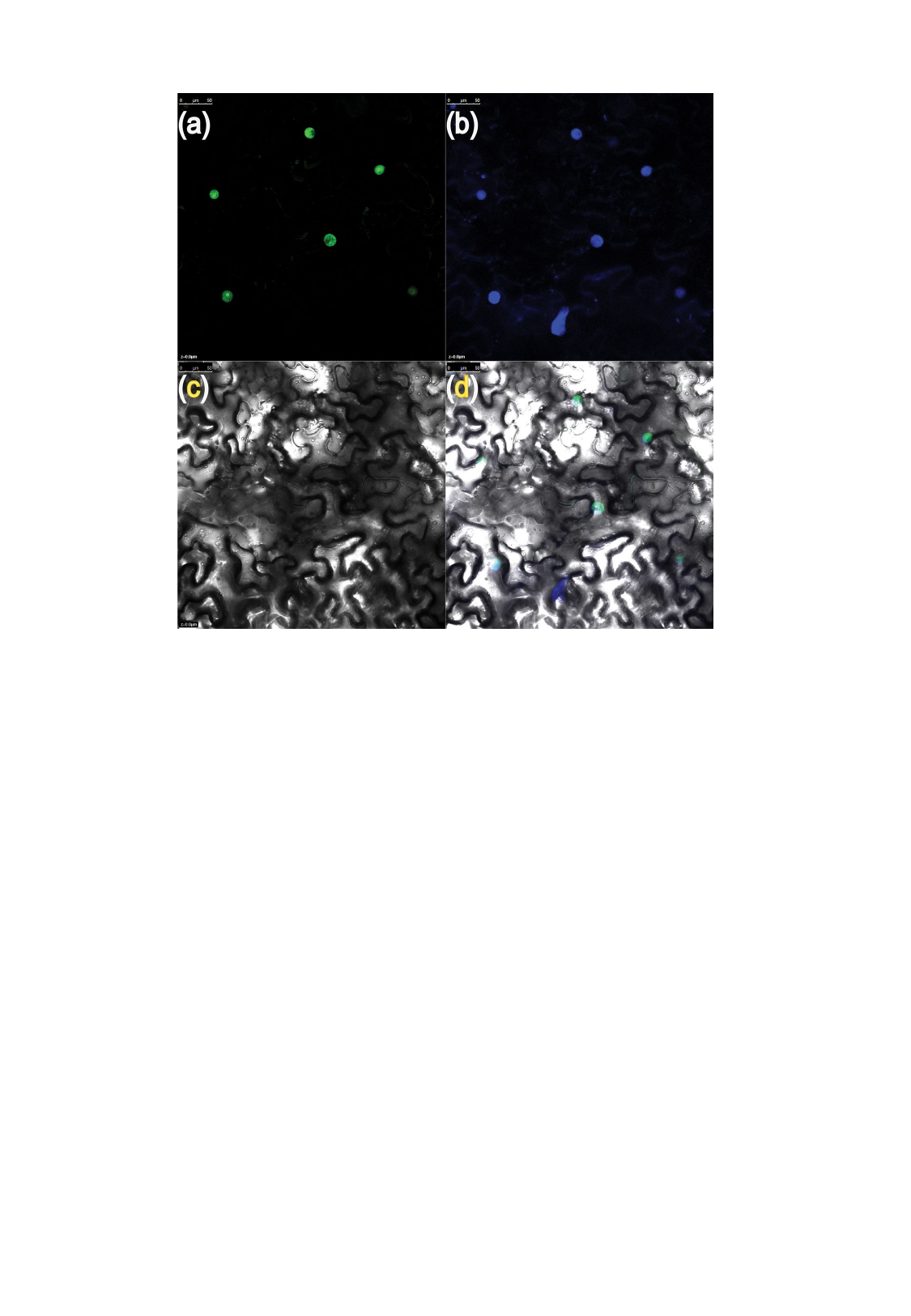

Supplement: Supplementary file 9 — Additional file 9: Fig. S9. GPLα-GFP is localized to the nuclei. Leaves were transiently transformed with a vector including a translational fusion between GPLα and GFP, 35S:GFP::GPLα. GFP protein localization was visualized using confocal microscopy. (a) GFP fluorescence; (b) DAPI nuclei staining dye fluorescence; (c) bright-field image; (d) overlay of a, b, and c. [file 12915_2021_1015_MOESM9_ESM.pptx]

## Slide 1
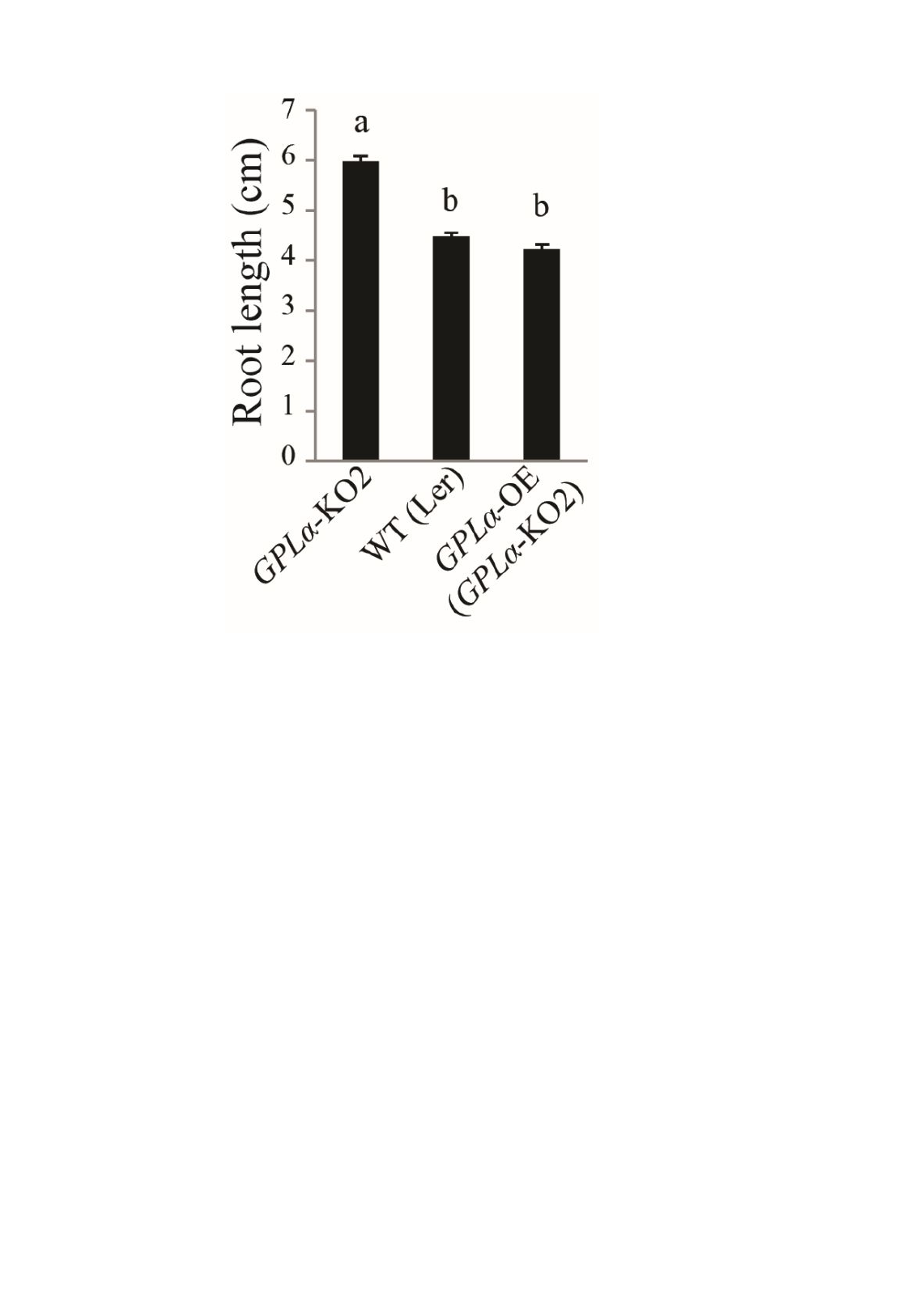

Supplement: Supplementary file 10 — Additional file 10: Fig. S10. Measured root length for the mutant line GPLα–KO2 in which GPLα was overexpressed [GPLα–OE (GPLα–KO2)]. The mutant line GPLα–KO2 (Ler background) was transformed for overexpression of GPLα and the consequences for root length following exposure to growth under Pi deficiency were examined in three independent transgenic lines. Different letters above the columns indicate significant differences (P < 0.05, ±SD). WT, wild type. [file 12915_2021_1015_MOESM10_ESM.pptx]

## Slide 1
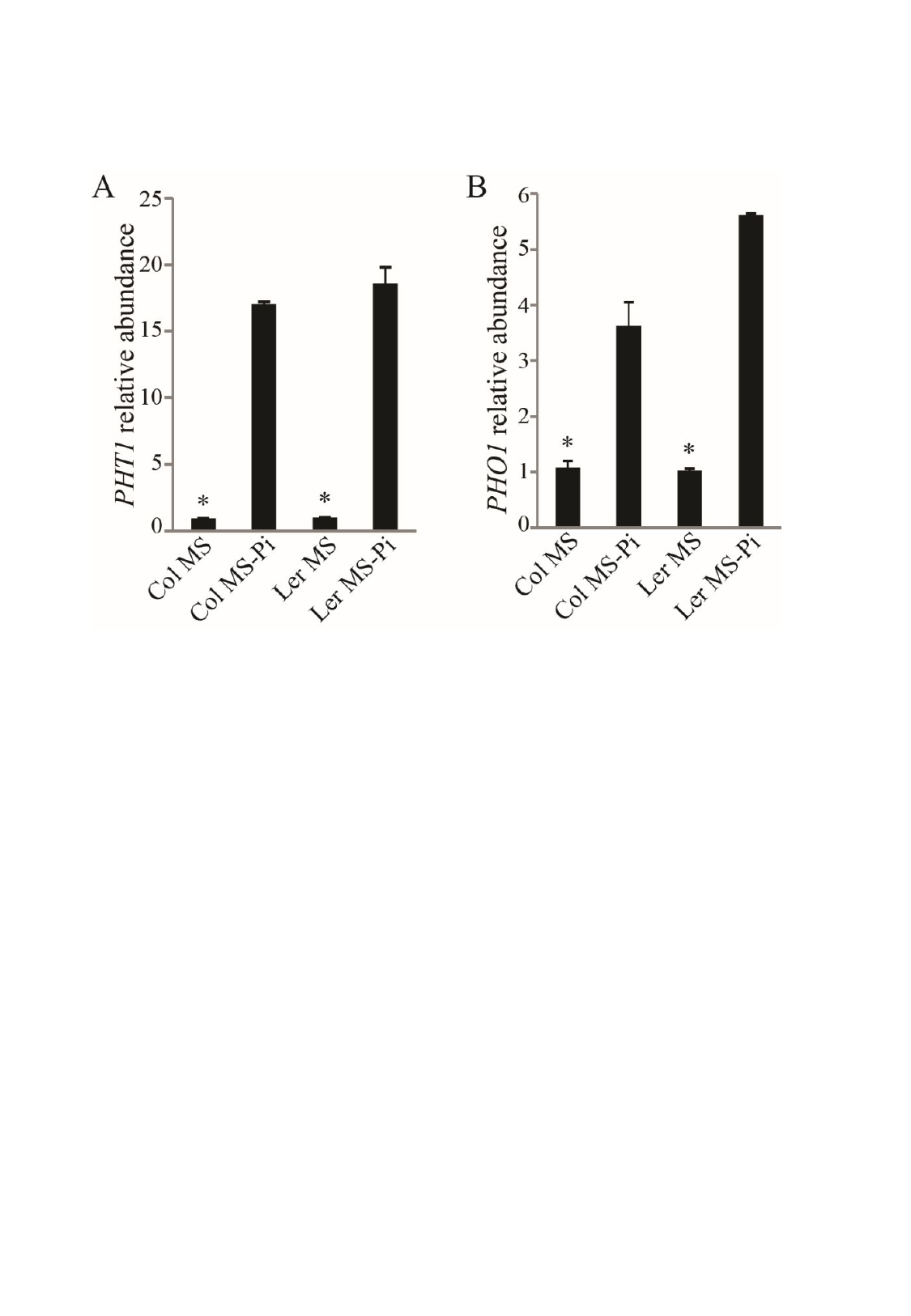

Supplement: Supplementary file 11 — Additional file 11: Fig. S11. Expression of PHT1 and PHO1 is induced under Pi-deficient conditions. Expression of PHT1 (A) and PHO1 (B) was measured in both Col-0 (Col MS) and Ler (Ler MS) wild-type accessions following transfer of seedlings to Pi-deficient growth conditions for 7 days (Col MS-Pi and Ler MS-Pi, respectively). Expression was measured by qRT-PCR and represents the mean of three biological repeats. Asterisks indicate significant difference (P < 0.05, Student’s t-test, ±SD). The primers used to measure PHT1 expression were as described for Additional file 8. [file 12915_2021_1015_MOESM11_ESM.pptx]

## Slide 1
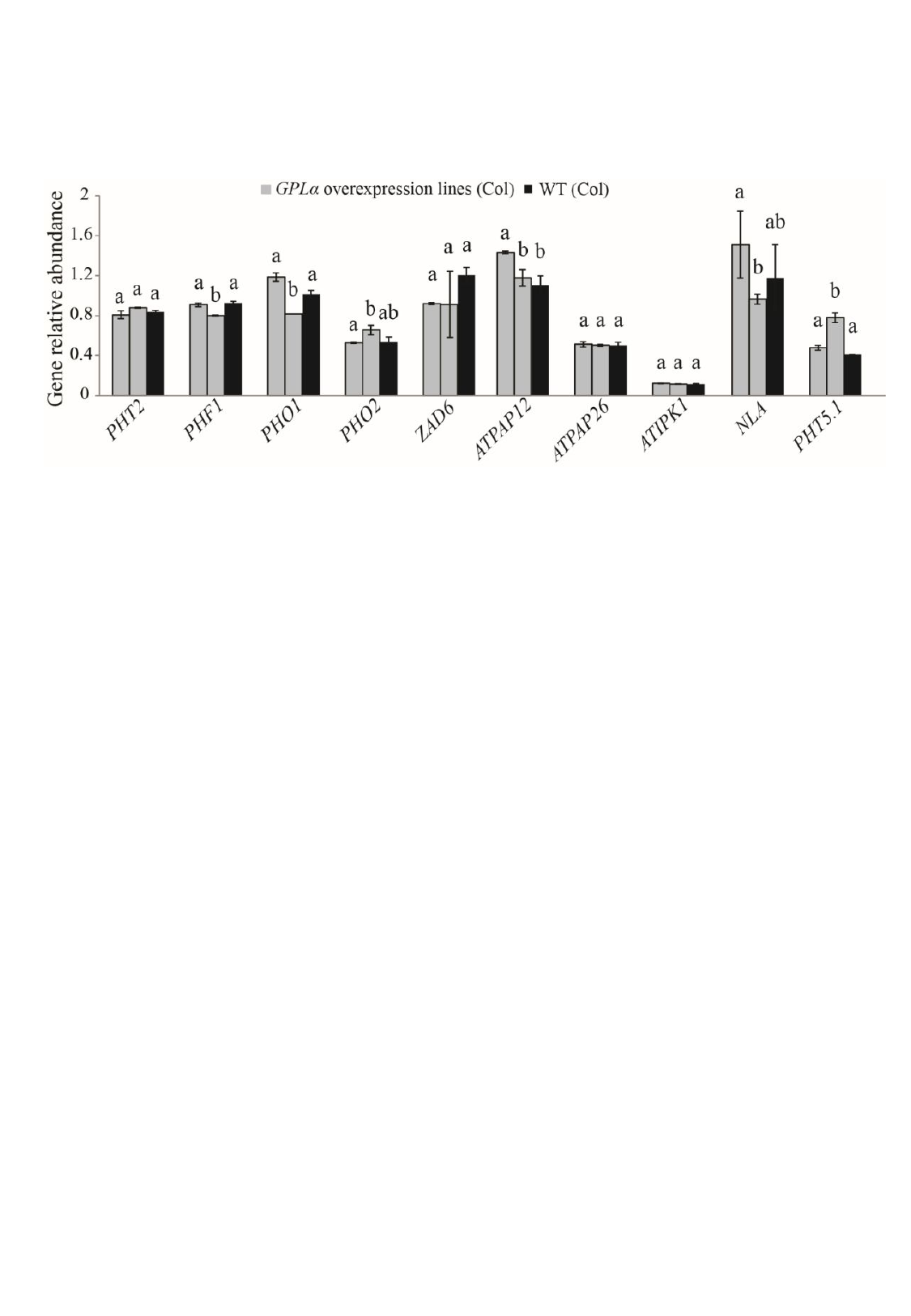

Supplement: Supplementary file 12 — Additional file 12: Fig. S12. Expression analysis of Pi-deficiency-responsive genes in GPLα-overexpressing lines. Expression was measured by qRT-PCR and represents the mean of three biological repeats. Different letters above the columns indicate significant differences (P < 0.05, ±SD). WT, wild type. [file 12915_2021_1015_MOESM12_ESM.pptx]
