# Supplementary material for: Downregulation of GeBP-like α factor by MiR827 suggests their involvement in senescence and phosphate homeostasis
Source: BMC Biol. 2021 May 3;19:90. doi: 10.1186/s12915-021-01015-2 (PMC8091714; doi:10.1186/s12915-021-01015-2)
Supplement: Supplementary file 13 — Additional file 13: Table S1. Sequences of primers used in gene cloning and vector construction. Table S2. Primers used for qRT-PCR analysis of gene expression. [file 12915_2021_1015_MOESM13_ESM.docx]

**Additional file 13: Table S1.** Sequences of primers used in gene cloning and vector construction

| **Gene primer name** | **Primer sequence (5' to 3')** |
| --- | --- |
| *ath-MIR827* | F: ATCTTGAGCTGCTTTGAGGAC |
| R: AGCCAGTAGGAATCCTGTCAA | |
| *ath-MIR827-1* | F: GGTCTAGAATCTTGAGCTGCTTTGAGGAC (*Xba*I) |
| R: GGCTCGAGAGCCAGTAGGAATCCTGTCAA (*Xho*I) | |
| *DBS* (GPL-FL) | F: ACACCATAAACCCCGTAAA |
| R: AAGCCATAAAGGTTCCAACAC | |
| *DBS* (GPL-FL-1) | F: TTCCATGGACACCATAAACCCCGTAAA (*Nco*I) |
| R: AATCTAGAAAGCCATAAAGGTTCCAACAC (*Xba*I) | |
| 827-STTM | F: AACCATGGAGTTTGTTGATCTA (*Nco*I) |
| R: AATCTAGATTAGATGACCTAG (*Xba*I) | |
| GPL-SI-1 | F: TTCTCGAGTTTCCGAGGACATGAATGG (*Xho*I) |
| R: TTCCATGGTTGGCTTCACAAGAGCAGTAG (*Nco*I) | |
| GPL-SI-2 | F: AATCTAGATTTCCGAGGACATGAATGG (*Xba*I) |
| R: TTGGATCCTTGGCTTCACAAGAGCAGTAG (*Bam*HI) | |
| MiR827-Pro | F: ATGTCGACCTAAGCAGCAGATTGAGGTGGTGG (S*al*I) |
| R: ACGCGGCCGCTATAACGTTTCATGGAAGTTAAAGA (N*ot*I) | |
| GPL-Pro | F: ATGTCGACGGCTTGCCATGCGTGGAA (S*al*I) |
| R: GTGCGGCCGCACCGAAACCATATATTGC (*Not*I) | |
| 35S-GPL-GFP | F: GTGTCGACATGGTTTCGGTGCAAAACCT (*Sal*I) |
| R: CACTGCAGAAACATATTTAGCCATCAAAGATCTCATCTC (*Pst*I) | |
| 35S-GFP-GPL | F: CACCATGGTTTCGGTGCAAAACC |
| R: CTAAACATATTTAGCCATCAAAGATC | |
| 827-STTM-F | F: AACCATGGAGTTTGTTGATCTA (*Nco*I) |
| 827-STTM-R | R: AATCTAGATTAGATGACCTAG (*Xba*I) |
| ath-miR827-pGreen 62-Sk | F: CGGGATCCTATAGGTTTTTTTCTTT (*Bam*HI) |
| R: CGGAATTCGATGCTAAAAACATG (*EcoR*I) | |
| GPLα-pGreen 0800 | F: GCTCTAGAATGGTTTCGGTGCAA (*Xba*I) |
| R: CGGAATTCCTAAACATATTTAGCCA (*EcoR*I) | |
| GPLα | F: ACAGGTAAGAATGTTTCCGAGG |
| R: TTGGCTTCACAAGAGCAGTA | |
| miR171d-pGreen 62-SK | F: GGACTAGTTAGATACACGAGATATT (*Spe*I) |
|  | R: TGCACTGCAGAGAAGTAGGACGTGA (*Pst*I) |
| SCL15-pGreen 0800 | F: GCTCTAGAATCTCCCACCTTCCCAA (*Xba*I) |
| R: CCACTAGTAGCCTCTGACCTGTCCG (*Spe*I) | |
| SCL15 | F: CCTCTGACCTGTCCGCAATCTCT |
|  | R: CTATGTTCTCCAACTTCACCGCC |

F, forward primer. R, reverse primer. Restriction sites are underlined.

**Additional file 13: Table S2.** Primers used for qRT-PCR analysis of gene expression

| **Gene primer name** | **Primer sequence (5' to 3')** |
| --- | --- |
| ath-MIR827-RT | GTCGTATCCAGTGCAGGGTCCGAGGTATTCGCACTGGATACGACAGTTTGTT |
| ath-miR827FWD | CGGTTAGATGACCATC |
| Universal primer | GTGCAGGGTCCGAGGT |
| *GPL* | F: TGAGCCTTCCAGCGATTCA |
| R: TCGGCTCAGCTTCAGTGTCA | |
| *NLA* | F: TTGAAAACCGCAGAAGCAACT |
| R: CCTTTATAAACCCCATCCTCACG | |
| *PHT5.1* | F: GTGGGTTGGGATCAGCAAGA |
| R: GATTCTCAAAGGCACACAATCG | |
| *SAG12* | F: GTGTCTACGCGGATGTGAAG |
| R: CAGCAAACTGATTTACCGCA | |
| *BFN1* | F: CCACGAGGTTGATTCTTGTATTAGG |
| R: TGCTCCAGCTTCGGACAGA | |
| *PHO1* | F: TACCTCTCCATTGCGTTGAATTT |
| R: CCCTGAATCTCATAATTGTCTCGAT | |
| *PHO2* | F: GGCTCAGTCCCAGGTCACAA |
| R: TTCGGAAGATTTGCCTCCAA | |
| *PHT1* | F: TGCCTGAAACTGCCCGTTA |
| R: CGGCTGTGGCTTGTTTGAT | |
| *PHT1.1* | F: GCCATGACGAGAAATAATTATGT |
| R: TAACTTAAGGTCAACGAGCCAAT | |
| *PHT1.2* | F: AGCCATCATTGGAGCCTTC |
| R: ACCTTAGCCTTGTCTTGATT | |
| *PHT1.3* | F: CGAGGCTGAGGTTGATAAATGAT |
| R: CACACATCGCAAAACCAATGAC | |
| *PHT1.4* | F: GGTCCCAATAGTTTAGGTGAT |
| R: AGTTGCTAGAGACAAGGAGAA | |
| *PHT1.5* | F: CGCTCTTGTAGCTCGGAATAC |
| R: TCCACTTGAAGCACCTTAGACA | |
| *PHT1.6* | F: ACGTTATACATCATGGCAGGAATCAAT |
| R: AAGCTCCTCAAGTGATTTCCCATTAGT | |
| *PHT1.7* | F: CGCGGCTTCTGGAAAATTAG |
| R: TGGAGGATATCCATGCTCTGTCT | |
| *PHT1.8* | F: TTATCCGAAGTAAACCGTATGAGAA |
| R: AATACGTCACCAAGATTCCAGCAA | |
| *PHT1.9* | F: TGGAGCTGCAGGGAAGTTTG |
| R: ATCTGGAAAACCGTCCTCTTCAT | |
| *PHT2* | F: TTGCTGGTCTCCTCTCTTCCTT |
| R: CATCCATAGTAAGAAGCTACCTGTAAC | |
| *PHF1* | F: AGTGGAAAGAGTGGCAGATATATGC |
| R: CTCGAAGAATACGTATGCAGCTATCA | |
| *ATIPK1* | F: CAAGGTATTTCTAGCGGTGGTGAT |
| R: GTTGGAAGAAATCCGCATTTG | |
| *AtPAP12* | F: TCGTTCCTGACATTGGTGAAATA |
| R: CTTATGTGGCGTATGATACCGATT | |
| *AtPAP26* | F: GGTCTGGCTGGAAGGTTTACG |
| R: AGCTAGCTTCCCGAAATGCA | |
| *ZAT6* | F: CGGAGACGAAGAGGTGATGAG |
| R: CGGGAAGTCAAACCGGAGTT | |
| *ACTIN-Ath* | F: CACTGTGCCAATCTACGAGGGT |
| R: GAGCTGGTCTTTGAGGTTTCC | |
| *UBC* | F: GCGACTCAGGGAATCTTCTAAG |
| R: CATCCTTTCTTAGGCATAGCG | |
| *ACTIN-Nb* | F: TGAGATGCACCACGAAGCTC |
| R: CCAACATTGTCACCAGGAAGTG | |

F, forward primer. R, reverse primer.
